# Lithium‐Containing Hybrid SEI Layer Enabling High Mass Loading and Anode‐Less Sodium Metal Batteries

**DOI:** 10.1002/anie.202423090

**Published:** 2025-03-30

**Authors:** Li Xia, Liang Lin, Jiantao Li, Yinggan Zhang, Hongfei Zheng, Xiaoqing Chang, Jinding Liang, Baisheng Sa, Laisen Wang, Jie Lin, Dong‐Liang Peng, Khalil Amine, Qingshui Xie

**Affiliations:** ^1^ State Key Laboratory of Physical Chemistry of Solid Surface College of Materials Xiamen University Xiamen 361005 China; ^2^ Chemical Sciences and Engineering Division Argonne National Laboratory Lemont IL 60439 USA; ^3^ Pritzker School of Molecular Engineering The University of Chicago Chicago IL 60637 USA; ^4^ Contemporary Amperex Technology Co., Ltd Ningde 352100 China; ^5^ Multiscale Computational Materials Facility College of Materials Science and Engineering, Fuzhou University Fuzhou 350100 China

**Keywords:** Diffusion kinetic, High mass loading, Low N/P ratio, Sodium deposition behavior, Sodium metal anode

## Abstract

The continuous rupturing and rebuilding of unstable solid electrolyte interphase (SEI) layer during cycling would block Na^+^ diffusion and induce Na dendrite formation, ultimately limiting the practical application of high‐energy‐density sodium metal batteries. Herein, a hybrid SEI layer containing Li‐species is dexterously constructed on the surface of sodium metal anode. Li‐containing inorganic components (Li_3_N, LiF, and Li_2_CO_3_) are introduced to stabilize the Na/electrolyte interface and enhance the mechanical and diffusion kinetic properties of the SEI layer, which can reduce the side reactions and gas generation, regulate Na^+^ flux during cycling and promote rapid Na^+^ migration for uniform dendrite‐free Na deposition. As a result, the constructed Na symmetric cells achieve low overpotential and long cycle life of 5900, 1800, and 500 h at current densities of 3, 10, and 30 mA cm^−2^, respectively. Furthermore, the full cells paired with the Na₃V₂(PO₄)₃ cathode demonstrate high specific capacity and excellent cycle stability, even at an ultra‐high cathode loading of 39.3 mg cm^−2^ and a low N/P ratio (negative/positive electrode capacity ratio of 1.21).

## Graphical abstract

A lithium‐containing hybrid SEI layer is designed for the Na metal anode by incorporating inorganic components (e.g., LiF, Li₃N, and Li₂CO₃) from the SEI layer of a Li metal anode. This design leverages the advantages of the SEI layer formed on Li anodes, which has a higher Young's modulus and better electrochemical stability (due to the lower redox voltage window of Li metal) compared to that formed on Na anodes. Simulations and extensive characterizations reveal that, with the benefits of high ionic conductivity, enhanced Young's modulus, reduced interfacial side reactions, and uniform Na^+^ deposition, the system achieves robust electrochemical performance in both symmetric and full cells.

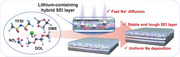

## Introduction

Severe environmental issues and nonrenewable resource shortages highlight the importance of high‐performance rechargeable batteries for green energy storage and conversion.^[^ ^1^ ^]^ A typical sign is that rechargeable lithium‐ion batteries (LIBs) have greatly reshaped modern lifestyles.^[^ ^2^, ^3^ ^]^ However, the growing demand for LIBs is causing lithium resources to become scarce and the price of lithium compounds to rise.^[^ ^4^, ^5^, ^6^ ^]^ Consequently, there is a strong incentive to develop low‐cost, high‐performance, and sustainable electrochemical systems, of which sodium‐ion batteries (SIBs) are considered a promising candidate.^[^ ^7^, ^8^, ^9^, ^10^ ^]^ Sodium metal as an anode for SIBs shows great advantages of high theoretical specific capacity (1166 mAh g^−1^), low redox potential (−2.71 V vs SHE), and abundant resources.^[^ ^11^, ^12^ ^]^ Unfortunately, several known fundamental obstacles exist for the practical application of sodium metal batteries (SMBs), including unstable solid electrolyte interphase (SEI) layer, uneven sodium deposition, and uncontrollable sodium dendrite growth.^[^ ^9^, ^13^, ^14^ ^]^ These factors are interrelated and will ultimately lead to rapid capacity fading of sodium batteries.

The electrochemical system maintains the stability of the anode‐electrolyte interface via the SEI layer. A stable SEI layer should be characterized by electronic insulation, high ion conductivity, and high mechanical strength.^[^ ^15^, ^16^, ^17^ ^]^ Ion transport in the SEI layer plays a critical role in charge transfer during cycling. Via a knock‐out mechanism, ion transport in the inorganic layer is shown to be the rate‐limiting step within the SEI layer.^[^ ^18^ ^]^ Sodium metal anode (SMA) cannot be effectively charged at high current densities (above 2 mA cm^−2^) due to severe side reactions that result in the decomposition of large amounts of electrolyte and formation of a thick SEI layer, and rapid Na^+^ deposition rates that far exceed diffusion rates, which can easily lead to the formation of dendrites.^[^ ^19^ ^]^ Moreover, the top deposition behavior of sodium metal severely strains the inherently inferior mechanical properties of the sodium‐based SEI layer.^[^ ^20^ ^]^ During electrodeposition, the mechanically unstable SEI layer is subjected to a large amount of stress and strain, easily leading to the fracture of the SEI layer and then dendrite infiltration.^[^ ^17^, ^21^ ^]^ In addition, exposing fresh sodium to electrolytes under this condition would result in both sodium and electrolyte depletion. Undoubtedly, degradation of the SEI layer due to mechanical and electrochemical/chemical instability would accelerate battery failure. Especially, under high cathode mass loading conditions and low N/P ratio, amplified volumetric expansion and polarization will pose significant challenges to the kinetic and mechanical performance of the SEI layer. Thus, increasing the ion transport kinetics and mechanical stability of the SEI layer on SMA is critical to suppress the drastic performance degradation, security threats, and failures in high‐energy‐density SMBs.

Strategies including electrolyte optimization, SMA interface modification, and other approaches have been used to modify the SEI layer.^[^ ^22^, ^23^, ^24^, ^25^ ^]^ NaF is the most common inorganic component of the SEI layer on SMA, which has the advantages of high Young's modulus (79.01 GPa) and excellent electronic insulation.^[^ ^26^ ^]^ However, in almost all cases, the diffusion barrier of NaF is ≈200 meV higher than that of LiF and its ionic conductivity is orders of magnitude less than LiF (≈10^−13^ vs ≈10^−7^ S cm^−1^).^[^ ^12^, ^27^ ^]^ In addition, the higher redox voltage of sodium anode with respect to SHE than that of lithium anode (−3.04 eV) indicates that the lithium‐based SEI layer may be more stable compared with the sodium‐based SEI layer in the same electrolyte potential window.^[^ ^16^ ^]^ Meanwhile, knock‐off mechanisms demonstrate that Li_2_CO_3_, the major inorganic component of the lithium‐based SEI layer, has great mechanical stability and a small Na^+^ diffusion barrier.^[^ ^18^, ^28^, ^29^ ^]^ When Na occupies an interstitial position, it can easily migrate through the LiF component with a low diffusion barrier by a direct hopping mechanism.^[^ ^27^, ^29^ ^]^ From the diffusion energy barrier point of view, direct hopping and knock‐off mechanisms in the lithium‐based SEI layer are the preferred pathways for Na^+^ migration. In addition, the SEI layer from the polished lithium (7.2 ± 0.7 GPa) shows a stronger Young's modulus than that from the polished sodium (≈1.3 GPa).^[^ ^30^ ^]^ Taking these into account, it is of great importance to introduce the effective components (e.g., LiF and Li_2_CO_3_) of lithium‐based SEI into the sodium‐based SEI layer to improve the electrochemical and mechanical interface stability of SMBs.

Herein, a lithium‐containing hybrid SEI (LSEI) layer is formed on the SMA surface by a simple, low‐cost electrochemical strategy (Table S1, Supporting Information). The lithium‐containing inorganics (LiF, Li_3_N, and Li_2_CO_3_) are introduced into the sodium‐based SEI layer using the lithium salt electrolyte. Such component modulation, taking the strengths of what is in the lithium‐based SEI layer, endows the sodium‐based hybrid SEI layer with high mechanical strength and rapid ion diffusion kinetics, and then guarantees uniform Na^+^ deposition and suppresses uncontrollable dendrite growth, reduces the side reaction and gas generation, finally extending the cycle life of both symmetric and full cells. Specifically, the constructed Na symmetric cell can cycle over 500 h at 30 mA cm^−2^ and 30 mAh cm^−2^. Meanwhile, the SMB matched with Na_3_V_2_(PO_4_)_3_ cathode can cycle for 1600 cycles at a large rate of 10 C, and a superior capacity retention of 97.9 % is harvested after 120 cycles even at an ultra‐high cathode mass loading of 39.3 mg cm^−2^. Besides, the anode‐less SMB with a low N/P ratio of 1.21 shows stable cycling performance with a high specific capacity of 99.6 mAh g^−1^ after 240 cycles. This work provides a promising strategy for developing high‐energy‐density and long‐life SMBs by regulating the SEI layer components.

## Results and Discussion

### Design and Characterization of the Lithium‐Containing Hybrid SEI Layer

**Figure 1:**
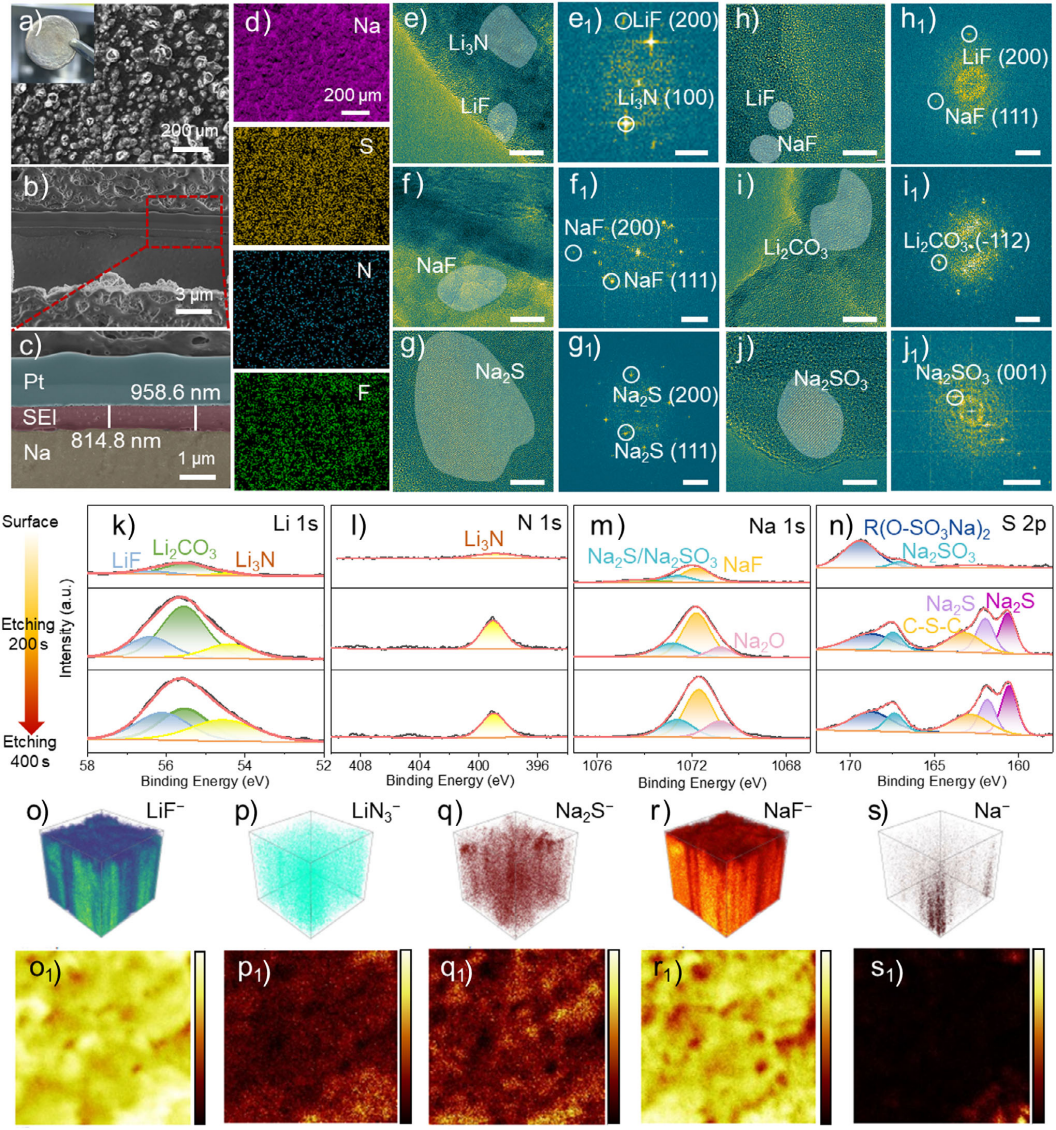
Component and structure characterization of LSEI layer. a) The top‐down SEM image of the LSEI layer, and the inset is the corresponding optical photograph. b,c) Cross‐section FIB‐SEM images of the LSEI‐Na. d) The elemental distribution mappings of Na, S, N, and F elements in the LSEI layer. High‐resolution TEM images (e–j, scale bar: 10 nm) and corresponding FFT images (e_1_–j_1,_ scale bar: 2.5 nm^−1^) of LiF, Li_3_N, NaF, Li_2_CO_3_, Na_2_S and Na_2_SO_3_. The high‐resolution XPS spectra of k) Li 1s, l) N 1s, m) Na 1s, and n) S 2p of the LSEI layer after different etching times of 0, 200, and 400 s. o–s) TOF‐SIMS 3D and o_1_–s_1_) top views reconstruction spectra of multiple secondary ion fragments of LiF^−^, LiN_3_^−^, Na_2_S^−^ NaF^−^ and Na^−^ on the LSEI‐Na surface.

A multifunctional lithium‐containing SEI (LSEI) layer is constructed on the SMA surface via a simple electrochemical strategy. Figures S1 and S2 (Supporting Information) show the preparation process of Na anode with a hybrid SEI layer with Li‐species (named LSEI‐Na). Scanning electron microscopy (SEM) and focused ion beam (FIB) were employed to observe the morphology and thickness of the constructed LSEI layer. As shown in Figure 1a, a large number of humps are distributed uniformly on the surface of LSEI‐Na. As shown in Figure 1b–d, a dense and homogeneous artificial SEI layer with an average thickness of ≈886.7 nm is formed on the SMA surface. The high‐resolution TEM images (Figure 1e–j; Figure S3, Supporting Information) show that the LSEI layer contains a variety of inorganic components, including Li_3_N, LiF, Li_2_CO_3_, NaF, Na_2_S, and Na_2_SO_3_, which are further confirmed by the corresponding fast Fourier transform (FFT) images. Specifically, as shown in Figure 1e and Figure S3a (Supporting Information), the lattice spacings of 3.08 and 2.03 Å represent the (100) plane of Li_3_N and the (200) plane of LiF, respectively. In addition, the high‐resolution TEM images (Figure 1i; Figure S3e, Supporting Information) show pronounced lattice fringes with lattice spacings of 2.61 Å, corresponding to the (−112) planes of Li_2_CO_3_. Therefore, X‐ray photoelectron spectroscopy (XPS) spectra of the LSEI layer were further tested to explore the specific component. The Li 1s, N 1s, Na 1s, S 2p, C 1s, O 1s, F 1s, and P 2p XPS spectra of the LSEI layer on the SMA surface after different etching times are collected and shown in Figure 1k–n and Figure S4 (Supporting Information). The Li 1s spectrum (Figure 1k) contains LiF (56.41 eV), Li_2_CO_3_ (55.50 eV) and Li_3_N (54.41 eV).^[^ ^26^, ^31^ ^]^ The N 1s XPS spectrum also proves the presence of Li_3_N (399.00 eV) in Figure 1l.^[^ ^32^ ^]^ The above results evidence that the lithium‐containing components have been successfully introduced into the SEI layer of SMA after electrochemical modification. Figure 1m displays the presence of four sodium‐containing components of Na_2_O (1070.80 eV), NaF (1071.80 eV), and Na_2_S/Na_2_SO_3_ (1072.81 eV) after etching.^[^ ^33^ ^]^ As shown in Figure 1n, the sulfur‐containing species are mainly derived from the related reaction products of lithium bis((trifluoromethyl)sulfonyl)azanide (LiTFSI) additive, including R(O−SO_3_Na)_2_ (168.79 eV), Na_2_SO_3_ (167.45 eV), C−S−C species (163.24 eV) and Na_2_S (160.65 and 151.99 eV).^[^ ^34^, ^35^ ^]^ After etching, four peaks are found in the C 1s spectrum at 284.80, 286.12, 289.08, and 290.30 eV, corresponding to C−C, C−O−C, COOR and CO_3_^2−^ groups, respectively (Figure S4a, Supporting Information).^[^ ^36^, ^37^ ^]^ There are two peaks in the F 1s spectrum at 684.73 and 685.43 eV, corresponding to NaF and LiF, respectively (Figure S4c, Supporting Information).^[^ ^26^, ^38^ ^]^ Notably, LiF and NaF, which are electrically insulating, have high ionic conductivity and strong mechanical strength.^[^ ^26^ ^]^ In view of the above, the LSEI layer consists of a combination of inorganic and organic phases. The organic components are produced by the decomposition of the electrolyte, while the inorganic components are formed through electrochemical reactions between Li/Na metal and electrolyte additives, such as LiNO₃ and LiTFSI. These inorganic phases, including LiF, Li_2_CO_3_, Li_3_N, Na_2_S, Na_2_SO_3_, NaF, and Na_2_O, are positioned closer to the Na metal surface, expected to inhibit dendrite growth and resist volume expansion during cycling.^[^ ^39^, ^40^ ^]^

The three‐dimensional distribution of different elements in vertical profiles on the LSEI‐Na anode was further investigated by the time of flight secondary ion mass spectrometry (TOF‐SIMS). As shown in Figure S5 (Supporting Information), the intensities of Li^−^, Na_2_O^−^, S^−^, SO_3_^−^, LiF^−^, LiN_3_^−^ and NaF^−^ secondary ion fragments gradually increase and remain stationary after 200 s etching, indicating that these fragments are mainly distributed within LSEI layer, corresponding to the inorganic components of LiF, Na_2_O, Na_2_S, Na_2_SO_3_, Li_3_N and NaF. Furthermore, the individual fragments are uniformly distributed in the 3D reconstruction and top view of the TOF‐SIMS spectra (Figure 1o–s and Figure S6, Supporting Information), indicating the good homogeneity of the components of the LSEI layer. Notably, the signal of the Na^−^ fragment almost cannot be observed in the 3D reconstruction and top view spectra (Figure 1s,s1), indicating that the electrochemically constructed LSEI layer completely covers the sodium metal surface, which benefits from reducing the interfacial side reactions between sodium metal and electrolyte.

### Electrochemical Performance and Deposition Behaviors of LSEI‐Na in Symmetric Cells

**Figure 2:**
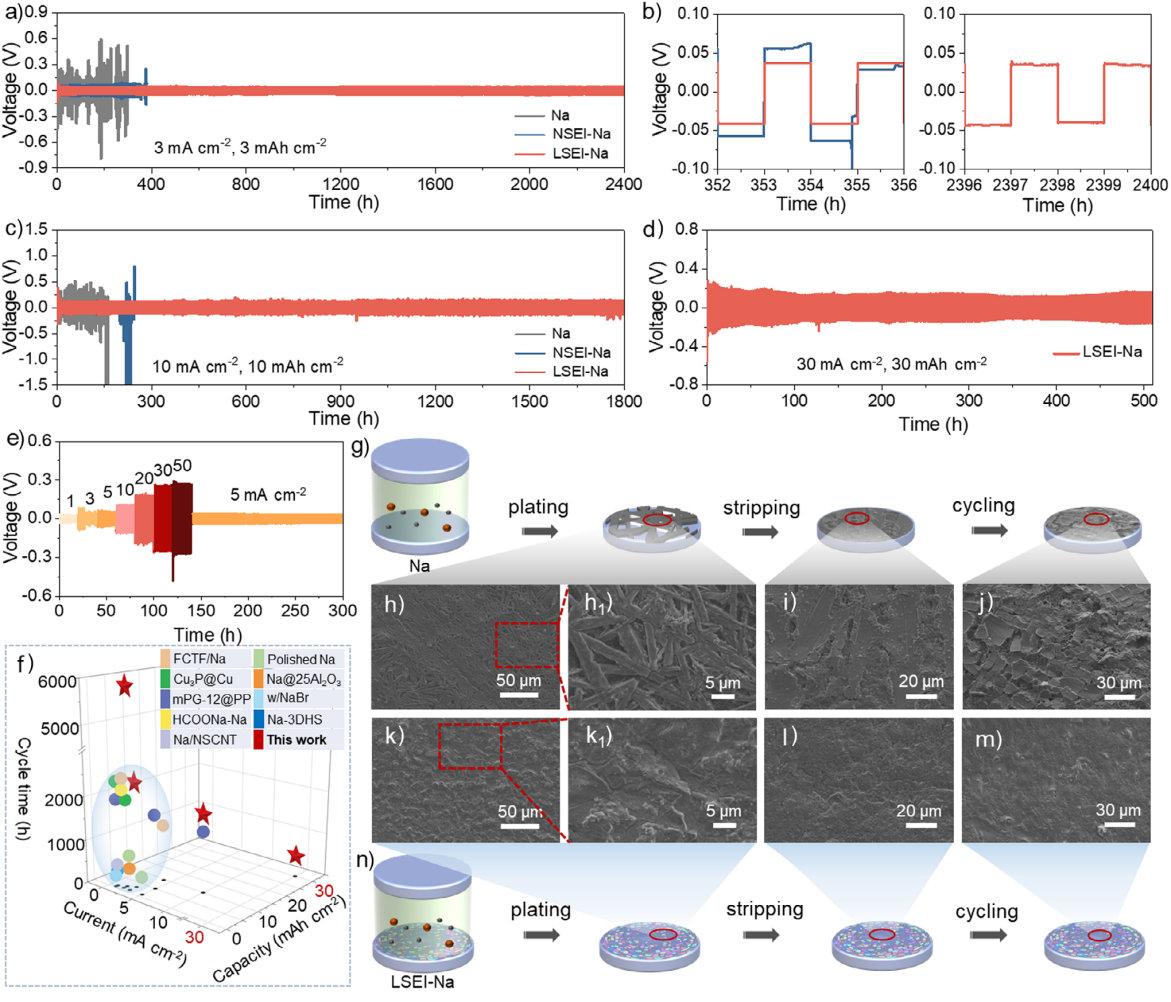
Electrochemical properties and deposition behaviors of LSEI‐Na symmetric cells. Cycling stability of symmetric cells with pristine Na, NSEI‐Na and LSEI‐Na anodes at a) 3 mA cm^−2^ and 3 mAh cm^−2^, c) 10 mA cm^−2^ and 10 mAh cm^−2^, and d) 30 mA cm^−2^ and 30 mAh cm^−2^. b) The time‐voltage curves at 3 mA cm^−2^ and 3 mAh cm^−2^ at different cycling time. e) Rate performance of LSEI‐Na symmetric cell with a fixed Na deposition for 1 h. f) The comparison of cycling performance between the previously reported works and this work. Schematic illustration of the symmetric cells with g) pristine Na and n) LSEI‐Na during cycling and the top‐down corresponding SEM images of symmetric cells by h, k) plating (1 mAh cm^−2^ at 0.5 mA cm^−2^), i, l) stripping (for 2 h at 0.5 mA cm^−2^) and j, m) cycling (50 cycles at 10 mA cm^−2^ and 10 mAh cm^−2^).

To demonstrate the crucial role of the LSEI layer in the electrochemical performance, different Na || Na symmetric cells with and without the LSEI layer were tested and compared. For comparison, the lithium salt electrolyte is replaced by the corresponding sodium salt electrolyte (1.0 m NaTFSI in DOL/DME with 2 wt% NaNO_3_ additive) in the electrochemical process to obtain an artificial SEI layer without lithium‐containing components (referred to as NSEI‐Na) (Figures S7, S8, Supporting Information). As shown in Figure S9 (Supporting Information), the SEI layer of NSEI‐Na is primarily composed of inorganic components such as NaF, Na₂SO₃, Na₃N, Na₂S, and Na₂CO₃, along with corresponding organic components. This composition differs from that of the LSEI layer, which includes additional Li species, such as Li₃N, LiF, and Li₂CO₃. The pristine Na, NSEI‐Na, and LSEI‐Na anodes were assembled into symmetric cells and initially tested at a current density of 3 mA cm^−2^ and a capacity density of 3 mAh cm^−2^. As exhibited in Figure 2a, the initial overpotential of the symmetric cell with pristine Na anode is 200 mV and surges to 600 mV only after cycling for 180 h, accompanied by a sharp voltage fluctuation, which is mainly due to the fracture of the formed native unstable SEI layer that would aggravate side reactions between sodium metal and electrolyte and thus hinder Na^+^ diffusion and resultantly induce uncontrollable Na dendrite growth. As shown in Figure 2a,b, a soft short‐circuit behavior occurs when the voltage of the NSEI drops suddenly at 355 h, and the overpotential gradually increases in the subsequent cycles, indicating that the fracture of the SEI layer would exacerbate the side reactions and dendrite growth. In sharp contrast, the LSEI‐Na symmetric cell exhibits an ultra‐long cycling performance of 2400 h with the lowest overpotential (≈40 mV) by means of the elaborately designed LSEI layer.

Since the currently reported artificial interfacial layers are practically incapable of achieving stable and long‐life SMA under harsh conditions (current densities greater than 10 mA cm^−2^) and SMBs at high cathode mass loading (greater than 10 mg cm^−2^), which severely hampers their commercial application.^[^ ^41^, ^42^, ^43^ ^]^ Therefore, the different symmetric cells are tested at higher current density and capacity (10 mA cm^−2^, 10 mAh cm^−2^). As shown in Figure 2c and Figure S10 (Supporting Information), both pristine Na and NSEI‐Na symmetric cells show large voltage polarizations over 1 V when cycled 169 and 220 h, respectively, while LSEI‐Na can cycle for 1800 h with a greatly reduced voltage polarization (0.125 V). Even at a very high current density of 30 mA cm^−2^ with a large deposition capacity of 30 mAh cm^−2^, the LSEI‐Na symmetric cell can be cycled more than 500 h (Figure 2d). As exhibited in Figure 2e, the LSEI‐Na symmetric cell is capable of cycling stably at a current density of up to 50 mA cm^−2^ with a lower overpotential of 270 mV, and can still stably cycle for hundreds of hours when the current density is reduced back to 5 mA cm^−2^. Therefore, the activation energy (E_a_) was tested to disclose the mechanism for the good cycling performance of the LSEI. Figure S11a–c (Supporting Information) shows the variable temperature electrochemical impedance spectroscopy (EIS) and corresponding fitted curves of Na, LSEI‐Na, and NSEI‐Na symmetric cells. Using the Arrhenius equation, the E_a_ of LSEI‐Na is calculated to be 10.0 kJ mol^−1^, lower than that of Na (15.4 kJ mol^−1^) and NSEI‐Na (14.7 kJ mol^−1^), suggesting that the LSEI layer has a superior Na^+^ transport ability and low Na^+^ diffusion barrier (Figure S11d, Supporting Information). The EIS test after cycling at high current density shows that LSEI minimizes the interfacial impedance during fast Na^+^ transport and shortens the gap between Na^+^ deposition rate and diffusion rate at high current density (Figure S12, Supporting Information). Such outstanding electrochemical performance of the LSEI‐Na symmetric cell is undoubtedly related to the introduced lithium‐containing components of the LSEI layer on the SMA surface, which will be discussed elaborately in the following part.

Subsequently, by changing the electrolyte composition, based on the electrochemical results, it can be found that LSEI‐Na anode delivers the highest stability and longest lifespan (cycling up to 5900 h at 3 mA cm^−2^ and 1 mAh cm^−2^), which is mainly attributed to the synergistic effect of the designed LSEI layer with multiple inorganic lithium/sodium components that can reduce side reactions and facilitate rapid Na^+^ diffusion, as analyzed in Figure S16 (Supporting Information) of the supporting information. From the above results, the LSEI‐Na symmetric cell has the most excellent cycle stability and long life compared to the previously reported works (Figure 2f and Table S5, Supporting Information).

The Na plating/stripping behaviors of pristine Na and LSEI‐Na anodes were investigated and schematically illustrated in Figure 2g,n. When deposited for 1 mAh cm^−2^ of Na at a current density of 0.5 mA cm^−2^, the pristine Na surface shows a mass of needle‐like Na dendrite (Figure 2h), whereas LSEI‐Na anode still possesses a flat and smooth surface (Figure 2k). Subsequently, after being stripped for 2 h, the surface of pristine Na shows large cracks and fragments (Figure 2i), while that of LSEI‐Na is quite smooth and well‐preserved (Figure 2l). When further cycled 50 times at 10 mA cm^−2^ and 10 mAh cm^−2^, the former native SEI layer could not endure the repeated plating/stripping at such conditions, as a result, the surface of pristine Na is severely destroyed and appeared obvious fragments (Figure 2j). Worse still, these fragments and cracks will accelerate the side reactions between sodium metal and electrolyte, leading to continuous thickening of the SEI layer and volume expansion, which ultimately retards Na^+^ diffusion. In contrast, the LSEI‐Na anode remains intact surface without crack formation after cycling (Figure 2m). Meanwhile, as revealed in Figure S17 (Supporting Information), the XPS spectra of the LSEI‐Na anode after cycling show that the components of the SEI layer are consistent with those before cycling. Namely, the introduced surface LSEI layer almost does not participate in the electrochemical reactions during cycling, successfully hindering disgusting side reactions. In‐situ optical microscopy is used to observe the influence of LSEI‐Na on the Na^+^ deposition behavior more directly. During the entire plating process at 1 mA cm^−2^, the Na is uniformly and dendrite‐free deposited on the LSEI‐Na surface (Figure S18a,b, Supporting Information). However, Na dendrites are detected on the pristine Na surface after 15 min of plating, and these dendrites become progressively larger as plating time increases (Figure S18c,d, Supporting Information), suggesting LSEI layer can effectively promote uniform Na deposition and inhibit the uncontrollable dendrite growth.

In addition, theoretical calculations further demonstrate the good sodiophilicity of the lithium‐containing inorganic components. As shown in Figure S19 (Supporting Information), Li_2_CO_3_, Li_3_N, and LiF have more negative adsorption energies than sodium metal for Na atoms absorption. Namely, Na atoms are more inclined to preferentially adsorb and deposit on the lithium‐containing components that are uniformly distributed in the LSEI layer, which is conducive to the formation of homogeneous and dense Na deposits. In addition, the nucleation overpotential of the symmetric cell also indicates that the LSEI layer shows stronger sodiophilicity than the natural SEI layer formed on the Na anode, which is favorable for the uniform deposition of Na^+^ (Figure S20, Supporting Information).

### Dynamics and Mechanical Properties of LSEI Layer

**Figure 3:**
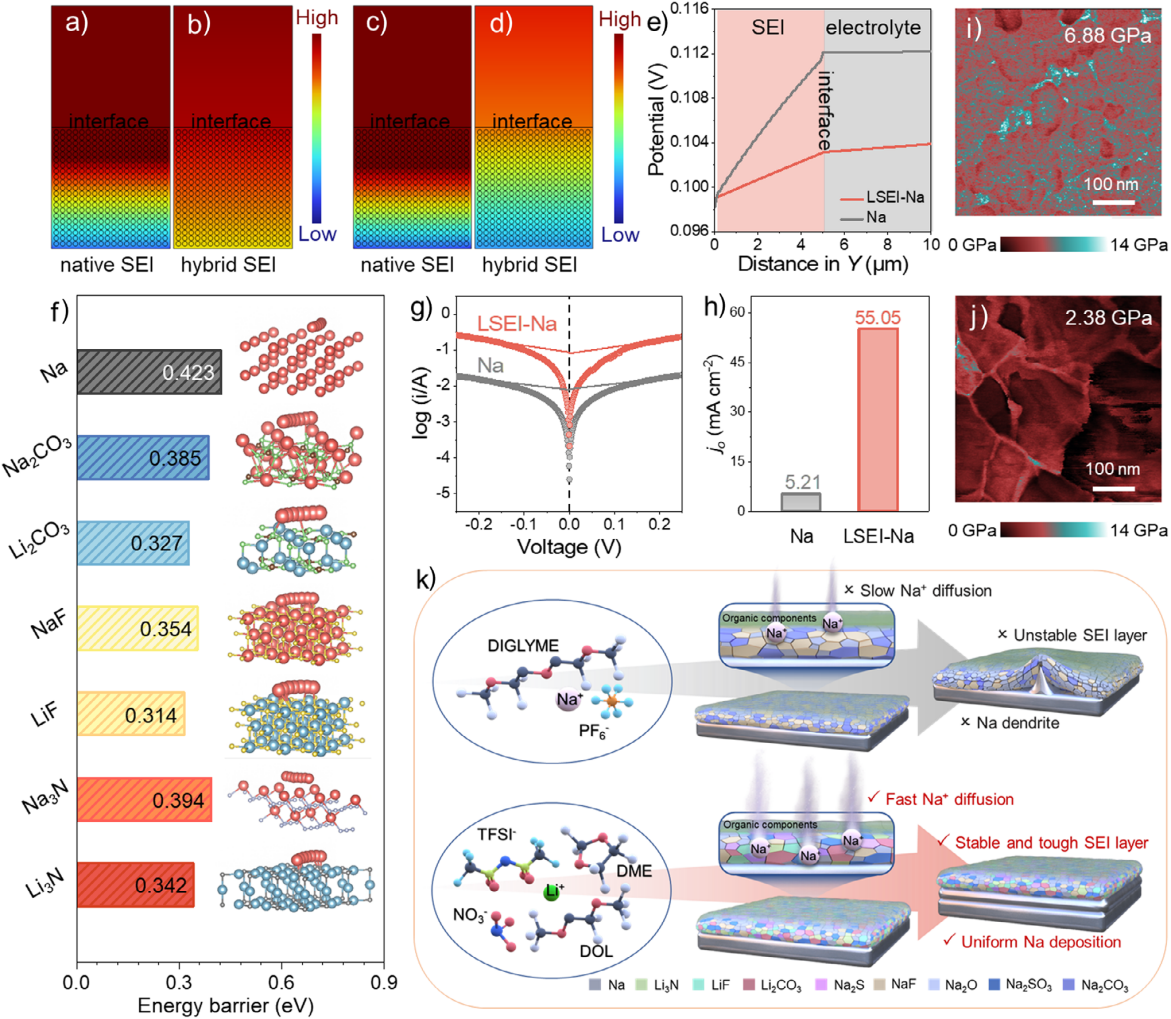
Dynamics and mechanical properties of LSEI layer. Finite element simulation of a,b) the Na^+^ concentration distribution and c,d) the electric field distribution of native SEI and LSEI layers. e) The cross‐sectional potential distribution along with *Y* direction in native SEI and LSEI layers. f) Diffusion energy and corresponding diffusion paths of Na^+^ in Na, Na_2_CO_3_, Li_2_CO_3_, NaF, LiF, Na_3_N and Li_3_N. g,h) Tafel curves of the symmetric cells of pristine Na and LSEI‐Na and the corresponding exchange current density. Young's modulus maps of i) LSEI‐Na and j) pristine Na anodes. k) Schematic illustrations of formation and deposition behavior on native SEI and LSEI layers.

To explore the intrinsic kinetic reasons for the uniform Na^+^ deposition endowed by the LSEI layer, the visualized analyses of the Na^+^ concentration and voltage distribution were carried out via finite element simulation (FES), and the diffusion barriers of Na^+^ in the LSEI layer were calculated using density functional theory (DFT). As shown in Figure 3a, there is an obvious gradient of Na^+^ concentration in the native SEI layer, indicating more Na^+^ aggregate at the interface and a lower Na^+^ concentration at the bottom. Due to the large Na^+^ concentration gradient, the insufficient supply of Na^+^ during plating leads to Na^+^ nucleation at the interface, ultimately exacerbating dendrite growth. Differently, the LSEI layer has a more uniform Na^+^ distribution (Figure 3b), which is conducive to uniform Na^+^ deposition using its excellent ion diffusion kinetics. The ability of LSEI‐Na to achieve a uniform Na^+^ distribution within the SEI layer is mainly due to the uniformly distributed ion‐conducting components in the LSEI layer (Figure 1o–s; Figure S6, Supporting Information). In‐situ EIS of the LSEI‐Na symmetric cell is shown in Figure S21 (Supporting Information). The impedance of the SEI layer (*R_SEI_*) remains unchanged during discharge, indicating that the LSEI layer is structurally stable and has consistent resistance to the Na^+^ transport, and further proves the uniformity of Na^+^ distribution within the SEI layer. The *R_ct_* exhibits a gradual increase during discharge, likely attributed to the concentration gradient at the electrode surface. In contrast, a significant reduction in *R_ct_* is observed upon charging, suggesting that cycling enhances interface activation, thereby optimizing the Na^+^ transport kinetics. It is observed from Figure 3c,d that the LSEI layer has a more even electric field distribution than the native SEI layer. The cross‐sectional voltage distribution along with the Y direction in Figure 3e shows a lower voltage gradient for LSEI‐Na than that of the pristine Na, which is responsible for the lower polarization voltage of the LSEI‐Na symmetric cell during cycling.^[^ ^44^ ^]^

In addition, the climbing‐image nudged elastic band method (CI‐NEB) is applied to calculate diffusion barriers of Na^+^ in Na, lithium‐containing components (Li_3_N, LiF and Li_2_CO_3_), and sodium‐containing components (Na_3_N, NaF, and Na_2_CO_3_). As shown in Figure 3f and Figure S22 (Supporting Information), the diffusion barriers of Na^+^ in Li_3_N, LiF, and Li_2_CO_3_ are lower than those of Na_3_N, NaF, and Na_2_CO_3_, respectively, demonstrating that lithium‐containing components are favorable for rapid Na^+^ diffusion. Meanwhile, the fundamental reason for electrochemical performance differences between LSEI‐Na and NSEI‐Na symmetric cells is also shown. In addition, Na_2_S and Na_2_SO_3_ have lower diffusion energy barriers (0.302 and 0.31 eV) as fast ionic conductors, which can improve the kinetics of the SEI layer (Figure S23, Supporting Information). The DFT calculations reveal that the Na^+^ diffusion barriers of each component in the LSEI layer are lower than that of Na (0.423 eV), which suggests that the LSEI layer can enhance Na^+^ migration. In Figure 3g,h, the LSEI‐Na possesses far higher exchange current density compared with pristine Na (55.05 vs 5.21 mA cm^−2^), proving that the LSEI‐Na has greatly enhanced ion diffusion kinetics. In addition, the Na^+^ transference number (t_Na_^+^) was used to assess the diffusion ability of Na^+^ in LSEI‐Na and pristine Na by the Vincent‐Bruce method. As shown in Figure S24 and Table S4 (Supporting Information), the t_Na_^+^ of LSEI‐Na is 0.88, which is higher than that of the pristine Na (0.69), evidencing its enhanced Na^+^ diffusion kinetics. As shown in Figure 3i,j, LSEI‐Na has a higher Young's modulus than pure Na (6.88 vs 2.38 GPa), which is beneficial to resist Na dendrite growth and reduces the risk of the SEI layer being poked by the dendrites as well as helps to resist the large volume change of SMA during cycling. The merits of the formed LSEI and native SEI layers as well as the corresponding sodium metal deposition behavior are schematically shown in Figure 3k. The native SEI layer, formed naturally by spontaneous reactions between sodium metal and electrolyte, has poor kinetics and low mechanical strength, whose structural integrity is easily compromised during repeated plating/stripping, exacerbating uneven charge distribution, reducing Na^+^ diffusion, inducing uncontrollable dendrite growth, and ultimately degrading the overall performance of the cell. In contrast, the LSEI layer with enhanced mechanical strength could promote rapid Na^+^ diffusion and achieve uniform and dendrite‐free sodium deposition.

### Application of LSEI‐Na Anode in Sodium Batteries

**Figure 4:**
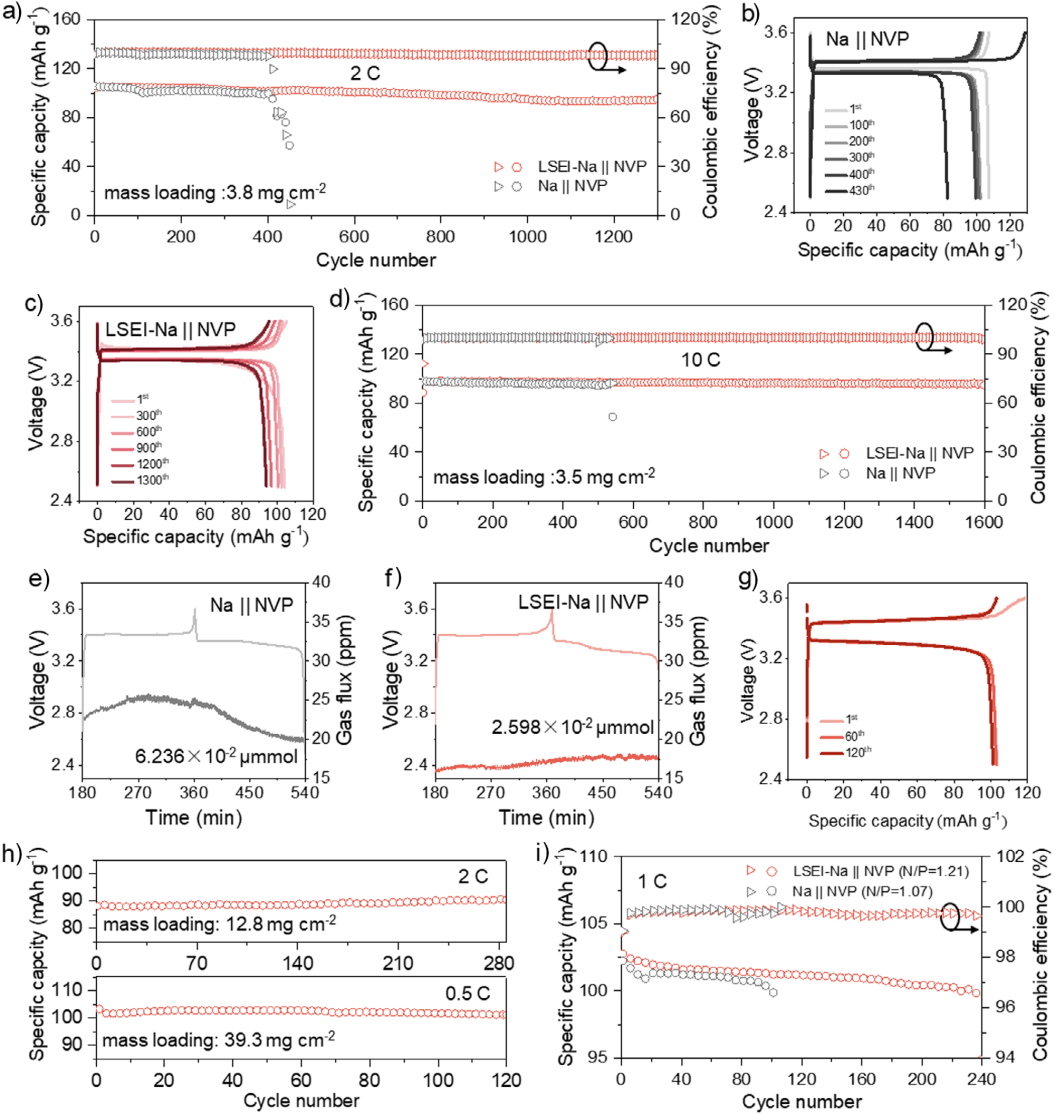
Electrochemical performance of LSEI‐Na || NVP full cells. a) Cycling performance and corresponding charge‐discharge curves of b) Na || NVP and c) LSEI‐Na || NVP full cells. d) Long‐life cycling performance at 10 C for Na || NVP and LSEI‐Na || NVP full cells. In‐situ DEMS tests for e) Na || NVP and f) LSEI‐Na || NVP full cells during the initial cycle (the amount of the released H_2_). g) The charge‐discharge curves of LSEI‐Na || NVP full cell at a high mass loading of 39.3 mg cm^−1^. h) Cycling performance of LSEI‐Na || NVP full cells at high mass loadings of 12.8 and 39.3 mg cm^−1^. i) The cycling performance of Na || NVP and LSEI‐Na || NVP full cells at low N/P ratio.

To assess the practical application of the designed LSEI‐Na anode, the full cells with LSEI‐Na anode and commercial Na_3_V_2_(PO_4_)_3_ (NVP) cathode were assembled and tested. As shown in Figure 4a, the discharge capacity of LSEI‐Na || NVP full cell is 95 mAh g^−1^ even after 1300 cycles at 2 C with an average coulombic efficiency (CE) as high as 98.9 %, while that of Na || NVP full cell decreases quickly and fades to 57.2 mAh g^−1^ after 450 cycles. In addition, it also can be observed through the corresponding charge‐discharge curves that Na || NVP full cell has been overcharged at 430th cycles, which is mainly due to uncontrollable Na dendrite growth and cathode structure deterioration (Figures 4b,c).^[^ ^45^ ^]^ Differently, LSEI‐Na || NVP full cell also exhibits long life and high cycle stability even at a very high current density of 10 C (Figure 4d; Figure S25, Supporting Information). After 1600 cycles, LSEI‐Na || NVP full cell has a discharge capacity of 94.7 mAh g^−1^ with a superior capacity retention of 97.3 % and an outstanding average CE of ≈100 %. In contrast, the discharge capacity of Na || NVP full cell fades abruptly to 68.8 mAh g^−1^ after 541 cycles. Clearly, the cycling stability of LSEI‐Na || NVP full cell is significantly superior to Na || NVP full cell, which is further confirmed by the gas generation experiment. Generally, the gas release takes place during SEI formation in SIBs. During the initial cycle, online continuous flow differential electrochemical mass spectrometry (DEMS) is used to measure the amount of gas released. As shown in Figure 4e,f, the amount of the released H_2_ in LSEI‐Na || NVP full cell is significantly reduced, indicating that the degradation of the electrolyte solvent is effectively inhibited.

In addition, LSEI‐Na || NVP full cells with high cathode mass loadings are also prepared and tested (Figure 4h; Figure S26, Supporting Information). The LSEI‐Na || NVP full cell with a high mass loading of 12.8 mg cm^−2^ exhibits excellent cycling performance with a capacity of 90.4 mAh g^−1^ at 2 C after 280 cycles. When the cathode mass loading is further increased to 39.3 mg cm^−2^, the LSEI‐Na || NVP full cell can still cycle stably for 120 cycles at 0.5 C with a high discharge capacity of 101.2 mAh g^−1^ (Figure 4g). To further determine whether the full cell failure is caused by negative electrode failure, the failed cell is disassembled and reassembled with a new electrolyte and cathode. As shown in Figure S27 (Supporting Information), the new full cell can still be cycled stably for 80 cycles, so it can be concluded that the failure of the cell is not due to LSEI‐Na anode rather than electrolyte depletion or collapse of the cathode structure. To gain a better understanding of the Na^+^ diffusion kinetics in the full cell, the Na^+^ diffusion coefficient was calculated using the galvanostatic intermittent titration technique (GITT) (Figure S28, Supporting Information). As shown in Figure S28c (Supporting Information), the diffusion coefficient of the LSEI‐Na || NVP full cell is higher than that of the Na || NVP full cell during cycling, demonstrating that the LSEI layer ensures fast transport of Na^+^ in full cell with high cathode mass loading. The fast Na^+^ diffusion rate in the LSEI layer prevents the concentration gradient on the positive side during charging and discharging caused by the insufficient supply of Na^+^, which exacerbates polarisation and leads to a reduction in the capacity of the cathode. Besides, the N/P ratio of the full cell is further controlled to evaluate the effect of the LSEI layer in high‐energy‐density SMBs. As shown in Figure 4i, the LSEI‐Na || NVP full cell with a low N/P ratio of 1.21, commonly used in commercial full cells, also exhibits remarkable cycling stability and delivers a high capacity of 99.6 mAh g^−1^ after 240 cycles, and this result opens up the possibility of achieving a high‐energy‐capacity SMBs. Meanwhile, the LSEI‐Na anode exhibits one of the best electrochemical performances, both in terms of long life, high mass loading, and low N/P ratio, compared to other recently reported interface engineering works for SMA (Table S6, Supporting Information).

## Conclusion

In conclusion, we propose a simple strategy to solve the problems of the instability, sluggish interfacial ion transport, and poor mechanical strength of the formed native SEI layer on the surface of the Na metal anode by introducing the inorganic components of the lithium‐based SEI layer. Comprehensive calculations and characterizations confirm that the hybrid SEI layer with Li‐species (e.g., LiF, Li_3_N, and Li_2_CO_3_) possesses excellent ionic conductivity, high Young's modulus, and good electrochemical stability, which can stabilize the Na/electrolyte interface, reduce gas generation and realize homogenous Na deposition without dendrite growth. As a result, the constructed symmetric and full cells exhibit enhanced cycle performance and high coulombic efficiency under stringent operating conditions and practical cell configuration with high cathode mass loading and low N/P ratio, showcasing their promising commercial viability. Further exploration of methods capable of large‐scale preparation of lithium‐containing component interfaces (e.g., electroplating, chemical coating, etc.) would facilitate the practical application of this strategy in SMBs.

## Conflict of Interests

The authors declare no conflict of interest.

## Supporting information



Supporting‐Information

## Data Availability

The data that support the findings of this study are available from the corresponding author upon reasonable request.
