# Supplementary material for: Lithium‐Containing Hybrid SEI Layer Enabling High Mass Loading and Anode‐Less Sodium Metal Batteries
Source: Angew Chem Int Ed Engl. 2025 Mar 30;64(21):e202423090. doi: 10.1002/anie.202423090 (PMC12087811; doi:10.1002/anie.202423090)
Supplement: Supplementary file 1 — Supporting‐Information [file ANIE-64-e202423090-s001.docx]

Supporting Information

Lithium-Containing Hybrid SEI Layer Enabling High Mass Loading and Anode-Less Sodium Metal Batteries

Li Xia,^+[a]^ Liang Lin,^+[a]^ Jiantao Li,*^[b]^ Yinggan Zhang,^[a]^ Hongfei Zheng,^[a]^ Xiaoqing Chang,^[a]^ Jinding Liang,^[d]^ Baisheng Sa,^[e]^ Laisen Wang,^[a]^ Jie Lin,^[a]^ Dong-Liang Peng,*^[a]^, Khalil Amine,*^[b],[c]^ Qingshui Xie*^[a]^

[*] L. Xia, L. Lin, Y. Zhang, H. Zheng, X. Chang, Dr. B. Sa, Dr. L. Wang, Dr. J. Lin, Prof. D. Peng, Prof. Q. Xie
State Key Laboratory of Physical Chemistry of Solid Surface, College of Materials, Xiamen University, Xiamen 361005, China
E-mail: dlpeng@xmu.edu.cn, xieqsh@xmu.edu.cn

Dr. J. Li,
Chemical Sciences and Engineering Division, Argonne National Laboratory, Lemont, IL 60439, USA
E-mail: jiantao.li@anl.gov

Prof. K. Amine
Chemical Sciences and Engineering Division, Argonne National Laboratory, Lemont, IL 60439, USA
Pritzker School of Molecular Engineering, The university of Chicago, Chicago, IL 60637, USA
E-mail: amine@anl.gov

J. Liang
Contemporary Amperex Technology Co., Ltd, Ningde 352100 China

B. Sa
Multiscale Computational Materials Facility, College of Materials Science and Engineering, Fuzhou University, Fuzhou, 350100, China.

[+] These authors contributed equally to this work.

**Experimental Section**

**Preparation of electrodes:** Firstly, asymmetric Na || Li cells (with Li as the cathode and Na as the anode) were assembled using a lithium-containing electrolyte consisting of 1.0 M LiTFSI in DOL/DME (1:1 by volume) with an additional 2 wt% LiNO₃. The asymmetric cell was then cycled under the following conditions: (I) plating 0.1 mAh cm^−2^ of Li at a current density of 0.5 mA cm^−2^, followed by a resting period of 1 hour; (II) galvanostatically cycling 50 times under 1.5 mA cm^−2^ and 0.1 mAh cm^−2^; (III) stripping 0.1 mAh cm^−2^ of Li at a current density of 0.5 mA cm^−2^. Finally, the asymmetric cells were disassembled and the SEI-coated Na plates were collected, washed with DOL/DME solvents and finally dried to obtain the sodium metal anode with lithium-containing hybrid SEI layer (LSEI-Na). To determine the effective composition for the formaiton of LSEI-Na, 1.0 M LiTFSI in DOL/DME (1:1 by volume) and 2 wt% LiNO₃ in DOL/DME (1:1 by volume) were used as electrolytes to form LiTFSI-Na SEI and LiNO₃-Na SEI on Na metal, respectively, following the same electrochemical procedures described above. For the synthesis of the SEI layer without lithium-containing inorganic components (NSEI-Na), a symmetric cell was assembled using an electrolyte composed of 1.0 M NaTFSI in DOL/DME (1:1 by volume) with 2 wt% NaNO₃ as an additive. The cell was then galvanostatically cycled 50 times at 1.5 mA cm^−2^ and 0.1 mAh cm^−2^. Finally, the symmetric cells were disassembled, and the SEI-coated Na plates were extracted, washed with DOL/DME solvents, and dried to obtain the NSEI-Na anode. The preparation of the sodium-based SEI layer (NP005-Na) is the same as for NSEI-Na, but the electrolyte is replaced with 1 M NaPF_6_ in diglyme. This sample is not mentioned in the manuscript, and it serves as the experimental basis for determining the specific components of the SEI layer of the pristine Na in the schematic illustration in Figure 3k. For the full cells with a low N/P ratio, 1 mAh of sodium was deposited onto the surface of the copper foil, and then subjected to the same electrochemical cycled step as for LSEI-Na. All of the above processes were carried out in a glove box filled with argon gas.

**Electrochemical testing:** All the cells were assembled using 2025-type coin cells in a glovebox filled with argon. The sodium foil used to assemble the cells was sourced from Changgao New Materials Co., Ltd, and the charge and discharge tests were conducted on the Neware cell testing systems. **The symmetric cells.** The symmetric cells were assembled with pristine Na, LiTFSI-Na, LiNO_3_-Na, NSEI-Na and LSEI-Na. The Celgard 2500 was served as the separator, and 1 M sodium hexafluorophosphate in diethylene glycol dimethyl ether (1 M NaPF_6_ in diglyme). Before the formal cycle, the cell was pre-activated at 0.25 mA cm^−2^ for 3 cycles, and cycle performance tests were carried out at different current densities. The electrochemical workstation was used to test the Tafel and LSV curves for the symmetric cells, with a voltage range from −0.25 V to 0.25 V and a scan rate of 1 mV s^−1^. **Na_3_V_2_(PO_4_)_3_ (NVP) full cell.** For the full cells, the Na_3_V_2_(PO_4_)_3_ (NVP) was applied as the cathode. The NVP cathode was prepared by mixing the NVP powder, acetylene black, and polyvinylidene fluoride (PVDF) with a weight ratio of 8: 1: 1 in N-methylpyrrolidone (NMP) to obtain a slurry. And then the slurry was fully stirred, coated on the carbon-coated Al foil and dried for 12 hours in a vacuum. Finally, the NVP cathode was cut into a disc with a diameter of 12 mm. The mass loadings of NVP were 3.5, 3.8, 8.7 and 12.8 mg cm^−2^. In addition, the NVP cathode with high mass loading (>20 mg cm^−2^) was prepared by mixing the NVP powder, acetylene black, and polytetrafluoroethylene (PTFE) with a weight ratio of 8: 1: 1 in isopropanol to obtain a slurry, which was rolled onto carbon-coated Al foil. The glass fiber (GF/D) and the Celgard 2500 were used as the separator, and 1 M NaPF_6_ in diglyme was employed as the electrolyte. The NVP full cells are cycled between 2.5−3.6 V under 0.5, 1, 2 and 10 C (1 C = 118 mAh g^−1^), respectively.

**Characterizations:** Field emission scanning electron microscopy (FESEM, Hitachi, SU-70) accompanied with x-ray energy spectroscopy (EDS) was used to observe the morphology and element distribution of the samples. Scanning Electron Microscope-Focused Ion Beam (FIB-SEM, Helios G4 CX) was used to prepare the samples for thickness investigation. The transmission electron microscopy (TEM, Talos F200s) was used to confirm the structure and phase compositions of LSEI. The chemical state of the material on the surface of the electrodes was studied by X-ray photoelectron spectroscopy (XPS, Thermo Scientific ESCALAB Xi+). Time of flight secondary ion mass spectrometry (TOF-SIMS) was employed to investigate the three-dimensional components of the samples. In-situ optical microscopy (Chongqingaote, SZ970) was applied to observe Na deposition behavior. The electrochemical impedance spectroscopy (EIS) tests were carried out on the electrochemical workstation with an amplitude of 5 mV and a 100 kHz~0.1 Hz frequency. All EIS tests were performed using 1 M sodium hexafluorophosphate in ethylene carbonate: dimethyl carbonate (1 M NaPF_6_ in EC:DMC) as electrolyte. To highlight the role of LSEI in sodium metal batteries, ester electrolyte was used for the interfacial impedance tests because the ester electrolyte is prone to decompose and generate a fragile SEI layer on the sodium metal surface, which leads to repeated SEI fracture/reconstruction and accelerated dendrite growth during cycling^[1]^. Young's modulus of the surfaces of electrodes was obtained using atomic force microscopy (AFM) in a glovebox filled with argon. The amounts of the released H_2_ during the initial cycle at 0.3 C between 2.5−3.6 V were evaluated by online continuous flow differential electrochemical mass spectrometry (DEMS). During GITT measurements, a short pulse of fixed current is applied (10 min), followed by a relaxation process (4 h) to obtain the voltage response over time (NVP mass loading ~24.25 mg cm^-2^).

**Calculation：** To verify the advantages of LSEI layer in regulating the Na^+^ concentration and potential distribution during Na^+^ deposition, the finite element simulations were performed on COMSOL Multiphysics 6.2. The steady-state LSEI and native SEI layers were plated in a 2D region of 5 × 10 μm^2^. The plating voltage difference between the two ends of the model was set as 0.2 V. The Na^+^ concentration was 1 M, and the Na^+^ diffusion coefficients in the native SEI and LSEI layers were 7.5×10^−14^ and 7.5×10^−12^ m^2^ S^−1^, respectively. The electrolyte conductivity was 0.5 S m^−1^. The exchange current densities of Na^+^ for the native SEI and LSEI layers were 0.521 and 5.505 A m^−2^, respectively. First-principles calculations based on density functional theory (DFT) were implemented in the Vienna *ab initio* Simulation Package (VASP)^[2]^. The energy cutoff for the plane wave was set to 520 eV. The climbing-image nudged elastic band method (CI-NEB)^[3]^ is applied for computing decomposition barriers. The Brillouin zone integration is performed using 2×2×1 k-point sampling. The bulk structures, surface structures and calculation models were dealt with the ALKEMIE platform^[4]^.

**
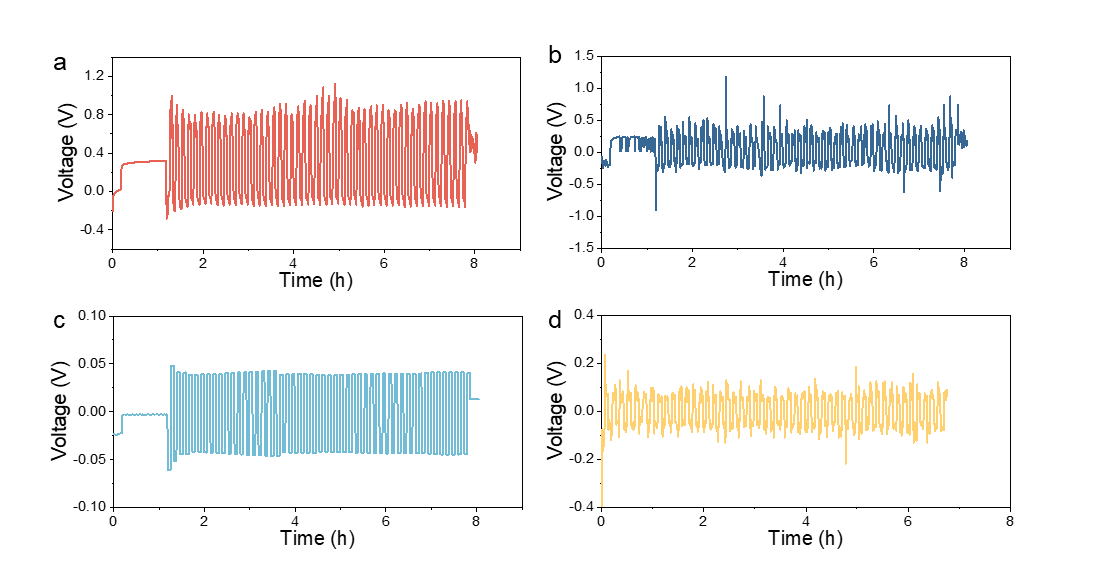
**

**Figure S1.** The time-voltage curves of the pre-cycling process of a) LSEI-Na, b) LiTFSI-Na, c) LiNO_3_-Na and d) NSEI-Na anodes.


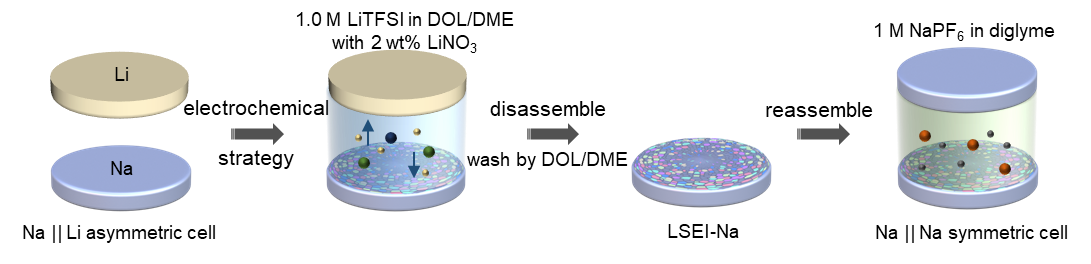


**Figure S2.** Schematic illustration of the per-cycling strategy for the synthesis of lithium-containing hybrid SEI layer.

Initially, the Na || Li asymmetric cell was assembled and electrochemically cycled for 50 times at a current density of 1.5 mA cm^−2^ using the electrolyte of 1.0 M LiTFSI in DOL/DME with 2 wt% LiNO_3_ additive (Figure S1a). Afterward, the Na || Li asymmetric cell was disassembled and Na anode with lithium-containing hybrid SEI layer (namely LSEI-Na) was collected after being washed with DOL/DME solvents. Finally, the Na || Na symmetric cell was assembled with LSEI-Na and 1 M NaPF_6_ in diglyme.


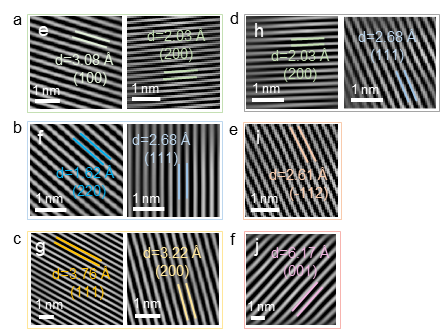


**Figure S3.** High-resolution TEM images in Figure 1e-1j corresponding to Inverse a-f) Fourier Transform Algorithm images of LiF, Li_3_N, NaF, Li_2_CO_3_, Na_2_S and Na_2_SO_3_.


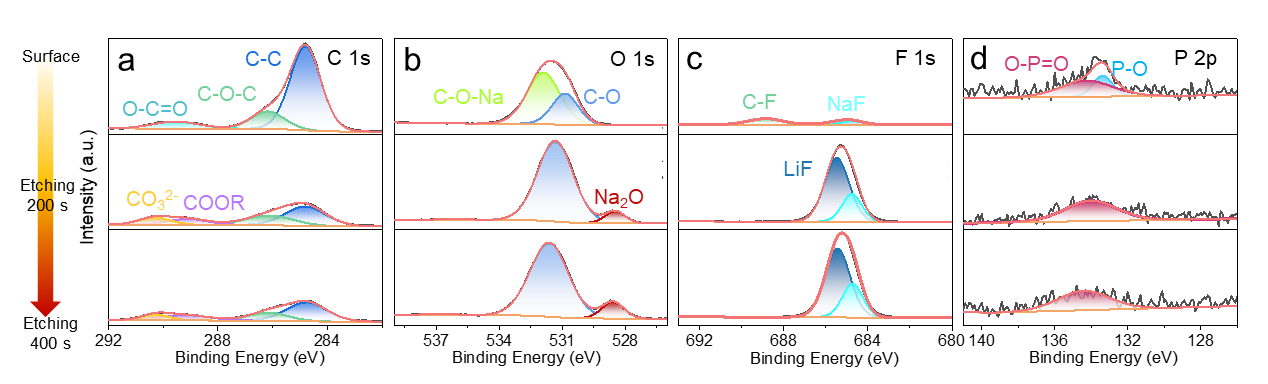


**Figure S4.** The high-resolution XPS spectra of a) C 1s, b) O 1s, c) F 1s and d) P 2p of the lithium-containing hybrid SEI layer after different etching time of 0, 200 and 400 s.


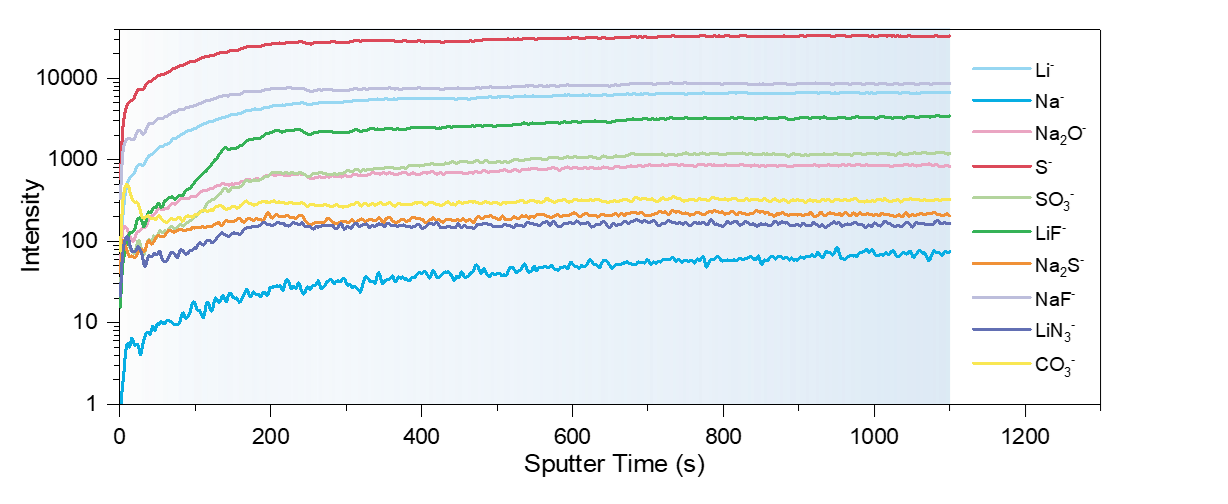


**Figure S5.** The intensity of multiple secondary ion fragments as a function of sputter time by time of flight secondary ion mass spectrometry (TOF-SIMS) on the surface of LSEI-Na. Sputter volume: 200🞨200 μm^2^.


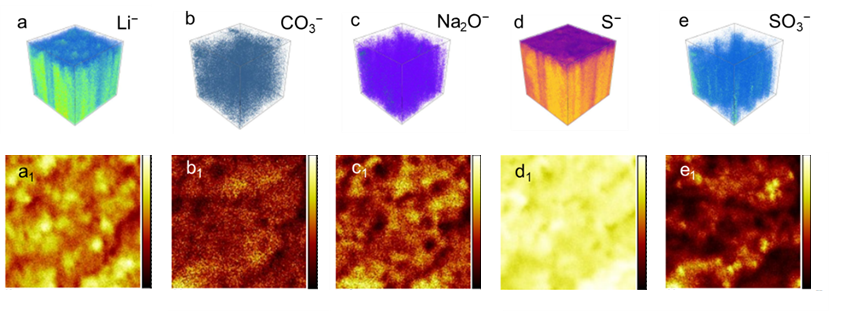


**Figure S6.** TOF-SIMS 3D and top views reconstruction spectra of several secondary ion fragments distributions of a) Li**^−^**, b) CO_3_**^−^**, c) Na_2_O**^−^**, d) S**^−^** and e) SO_3_**^−^** on the surface of LSEI-Na. Sputter volume: 200🞨200 μm^2^.


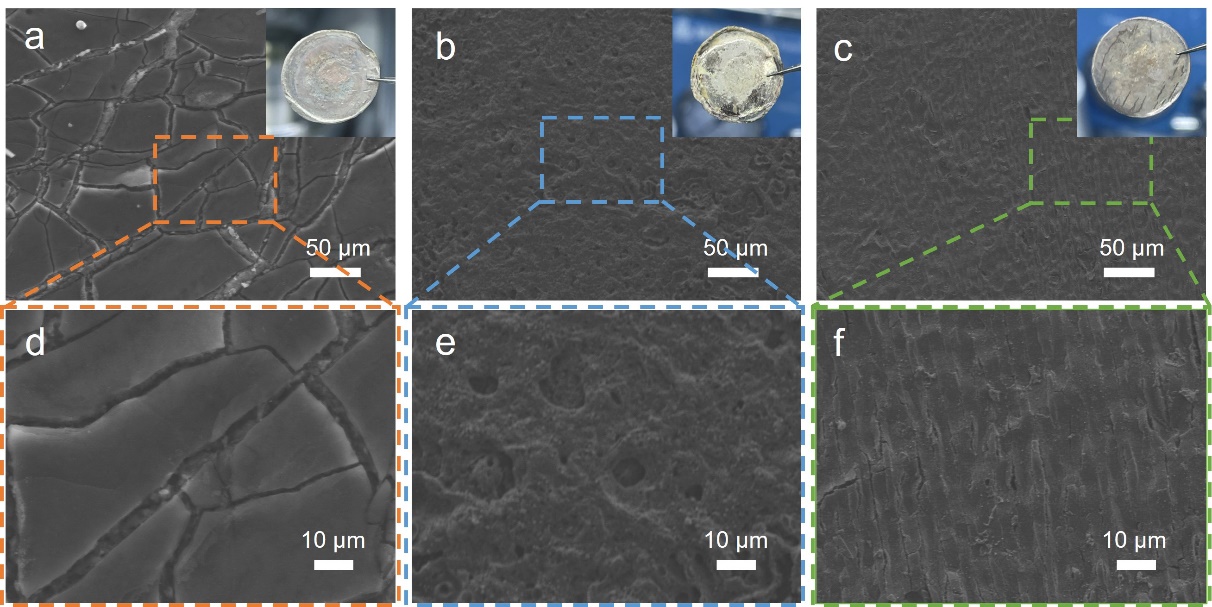


**Figure S7.** The SEM images and corresponding magnified images (the inset is the corresponding optical photograph) of a, d) LiNO_3_-Na, b, e) LiTFSI-Na and c, f) NSEI-Na anodes.


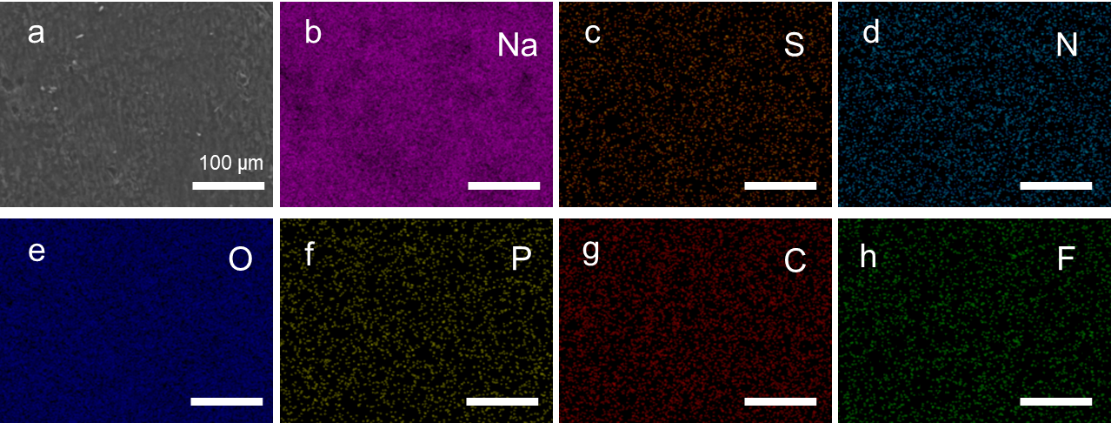


**Figure S8.** The SEM images and corresponding elemental distribution mappings of Na, S, N, O, P, C and F elements in NSEI-Na.


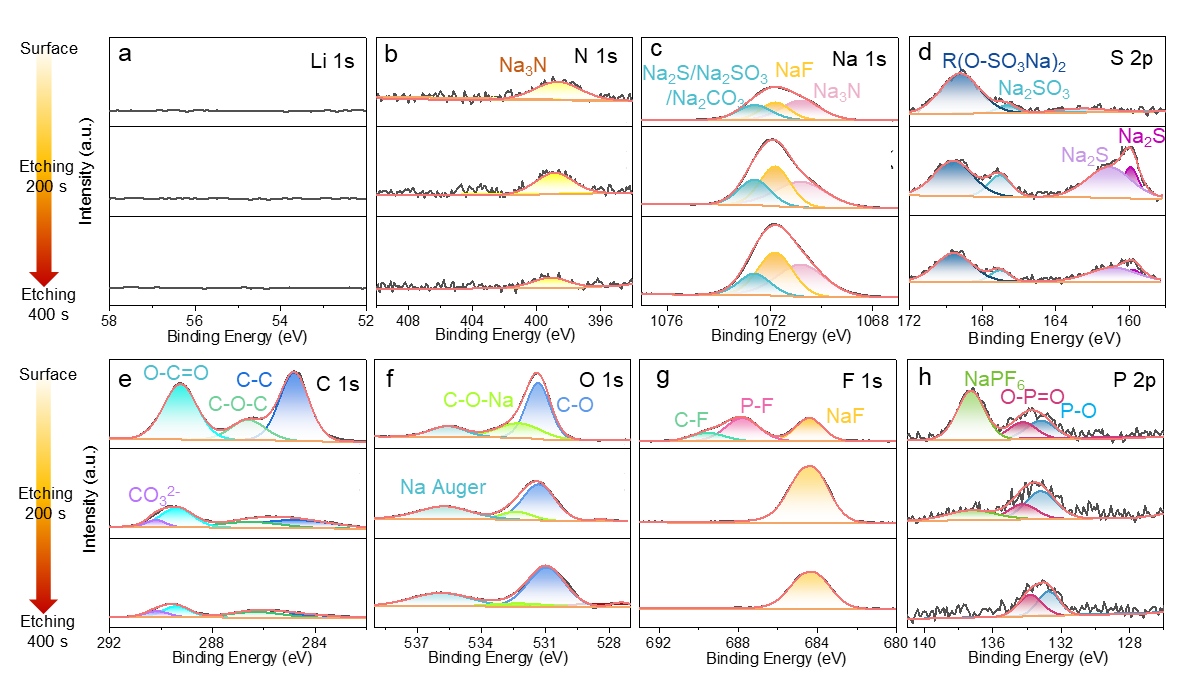


**Figure S9.** The high resolution XPS spectra of a) Li 1s, b) N 1s, c) Na 1s, d) S 2p, e) C 1s, f) O 1s, g) F 1s and h) P 2p of the SEI layer of NSEI-Na after different etching time of 0, 200 and 400 s.


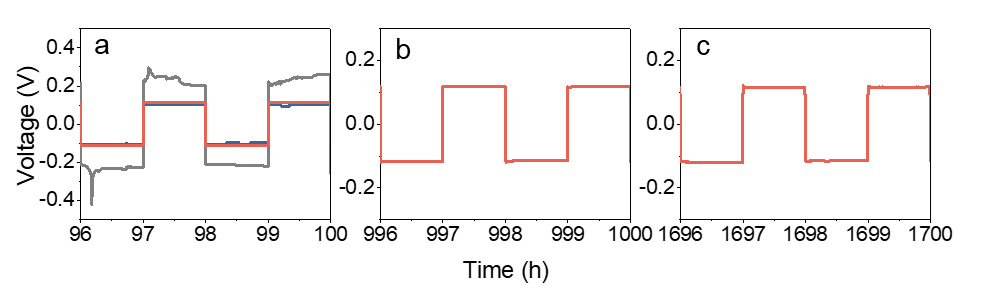


**Figure S10.** The time-voltage curves at 10 mA cm^-2^ and 10 mAh cm^-2^ at certain time from Figure 2c.


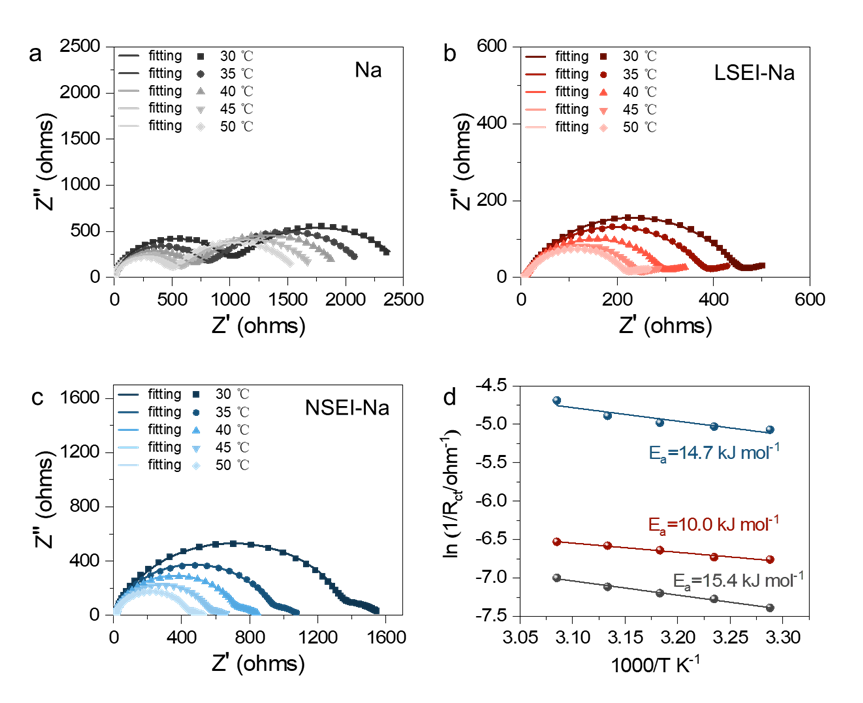


**Figure S11.** EIS spectra of symmetric cells with a) Na, b) LSEI-Na and c) NSEI-Na anodes at different temperatures. d) The calculated activation energies of Na, LSEI-Na and NSEI-Na anodes via the Arrhenius equation.


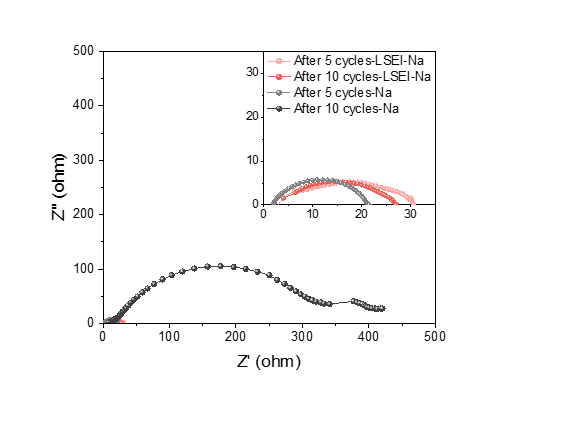


**Figure S12.** Electrochemical impedance spectroscopy of LSEI-Na and Na symmetric cells at different cycles under 10 mA cm^−2^ and 10 mAh cm^−2^.


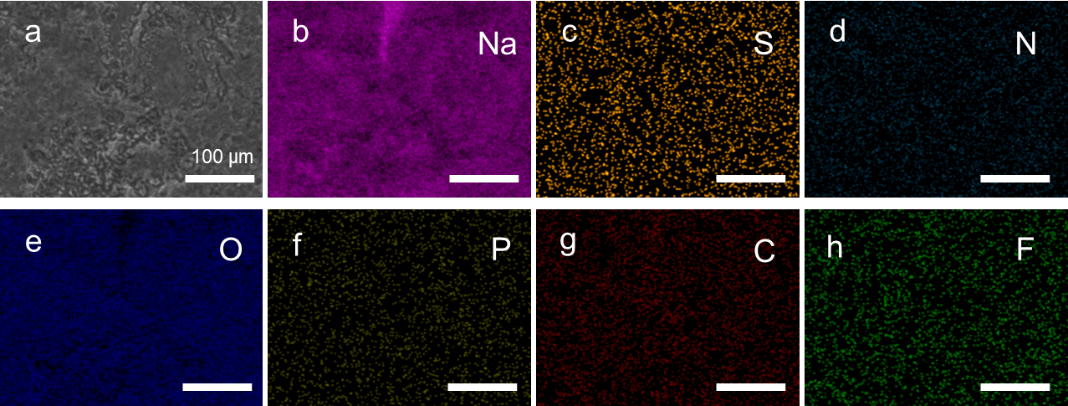


**Figure S13.** The SEM images and corresponding elemental distribution mappings of Na, S, N, O, P, C and F elements in LiTFSI-Na.


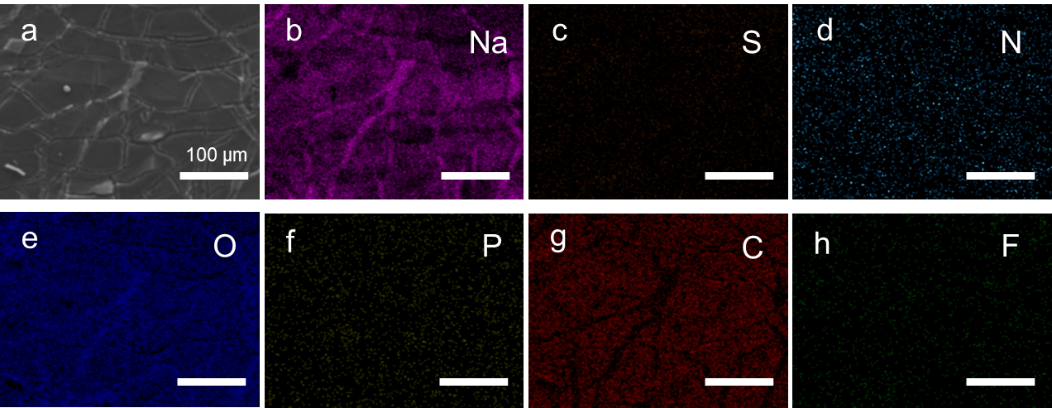


**Figure S14.** The SEM images and corresponding elemental distribution mappings of Na, S, N, O, P, C and F elements in LiNO_3_-Na.


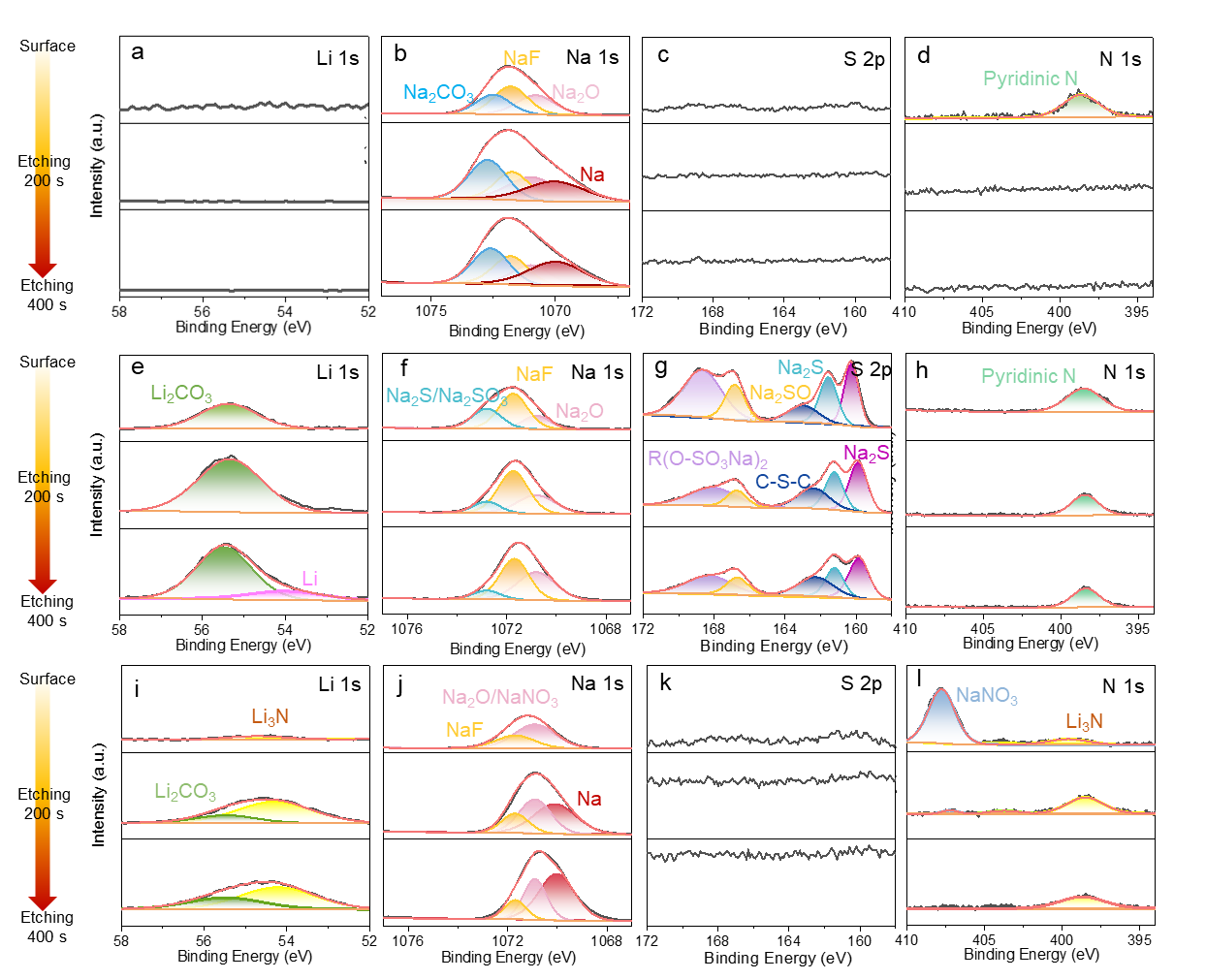


**Figure S15.** The high resolution XPS spectra of Li 1s, Na 1s, S 2p and N 1s of the SEI layers of a-d) NP005-Na, e-h) LiTFSI-Na and i-l) LiNO_3_-Na after different etching time of 0, 200 and 400 s.


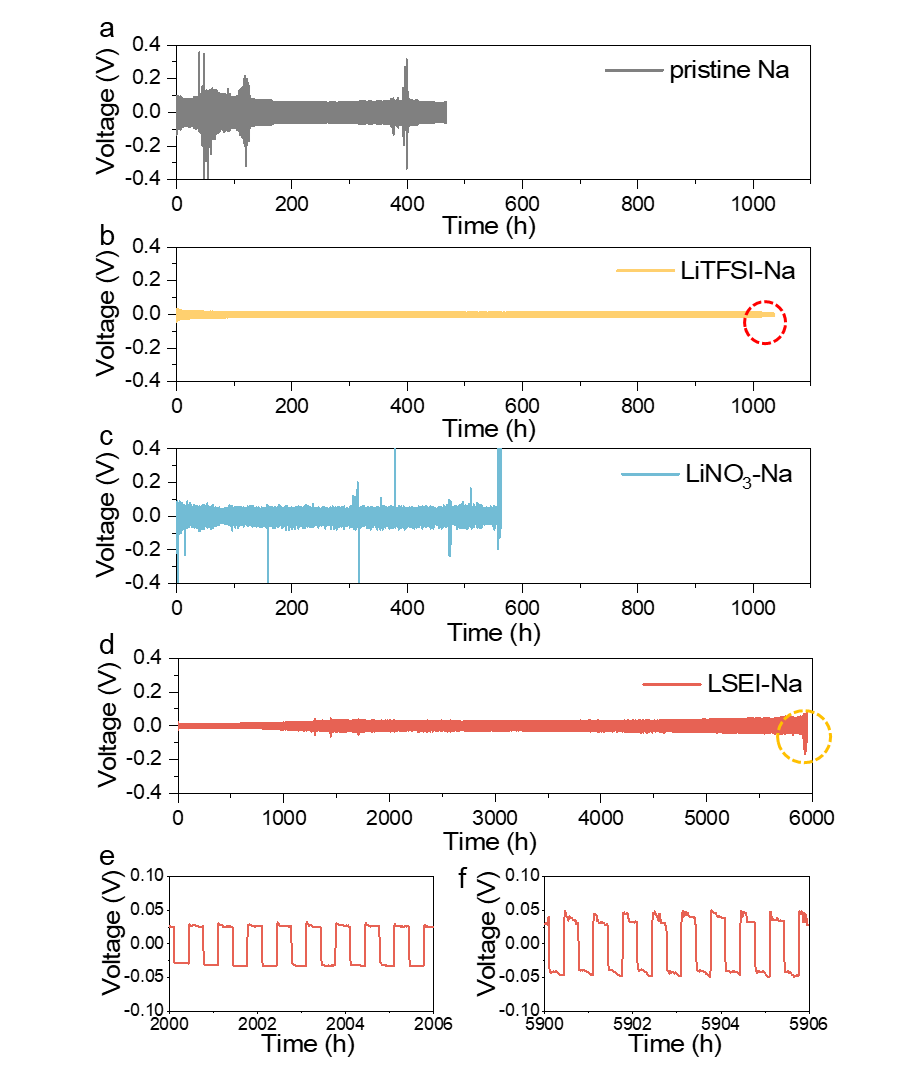


**Figure S16.** The cycling performance of symmetric cells with a) pristine Na, b) LiTFSI-Na, c) LiNO_3_-Na and d-f) LSEI-Na anodes at a current density of 3 mA cm^-2^ and a capacity density of 1 mAh cm^-2^.

To further explore the effect of the lithium-containing components in the LSEI layer on the electrochemical performance, a series of SEI layers with different lithium components were prepared by using different electrolytes in the same electrochemical process. For easy distinction, the anode obtained using 1.0 M LiTFSI in DOL/DME electrolyte and using 2 wt% LiNO_3_ in DOL/DME electrolyte are named as LiTFSI-Na and LiNO_3_-Na, respectively. The morphology and elemental distribution mappings of LiTFSI-Na and LiNO_3_-Na are demonstrated in Figures S7, S13 and S14. As shown in Figure S15, the SEI layer of LiTFSI-Na anode mainly consists of Li_2_CO_3_, Na_2_S, NaF, Na_2_O, Na_2_SO_3_, and that of LiNO_3_-Na anode consists of Li_2_CO_3_, Li_3_N, NaF, Na_2_O. The symmetrical cells were assembled with pristine Na, LiTFSI-Na, LiNO_3_-Na and LSEI-Na anodes, and tested at 3 mA cm^−2^ and 1 mAh cm^−2^ (Figure S16). As shown in Figure S16a and c, the time-voltage curves of symmetric cells of pristine Na and LiNO_3_-Na are very fluctuating and rough (pristine Na is worse) with a large voltage polarization close to 100 mV, and the LiNO_3_-Na symmetric cell cycles only about 500 h. The poor cycling performance may be attributed to continuous formation and accumulation of unstable SEI layer, leading to slow Na^+^ diffusion, sodium dendrite growth and cell failure. The symmetric cell with LiTFSI-Na can be cycled stably for 1000 h (Figure S16b). In contrast, the symmetric cell with LSEI-Na anode exhibits an ultra-long cycling stability of up to 5900 h with a lower voltage polarization of 50 mV (Figure S16d−f).

LSEI-Na anode reveals exceptional cycling stability (5900 h at 3 mA cm⁻² and 1 mAh cm⁻²) than LiTFSI-Na, LiNO₃-Na, and LSEI-Na counterparts obtained from varied electrolyte compositions (Figure S16, Supporting Information), which is attributed to synergistic interactions among multiple inorganic components in the engineered LSEI layer. Through comparative analysis of different systems, the component-function relationships are elucidated and descripted as follows:

Na₂S and Na₂SO₃ can provide low energy barrier Na^+^ transport channels, improve the Na^+^ diffusion kinetics and reduce the interfacial impedance. Li₂CO₃, which has high mechanical strength and ionic conductivity, and organosulfide with flexibility, can form a rigid-flexible composite structure that combines mechanical strength and deformation adaptability, thus resisting dendrite growth and reducing the risk of SEI layer fracture. NaF and Na_2_O domains, as the common components of sodium-based SEI layer, can stabilize the interface. Li₃N as a super-ionic conductor can significantly enhance the Na^+^ diffusion kinetics.

The SEI layer of LiTFSI-Na consists of inorganic components such as Li_2_CO_3_, Na_2_S, NaF, Na_2_O, Na_2_SO_3_. LiTFSI-Na symmetric cells achieve stable cycling with low polarisation potentials, but the SEI layer containing only Li_2_CO_3_ with high mechanical strength is unable to withstand the volume change during long-term cycling, which leads to the fracture of SEI layer during cycling and soft short-circuits occurring after 1000h cycling.

The LiNO₃-Na (Li_2_CO_3_, Li_3_N, NaF, Na_2_O) exhibits different behavior: LiNO₃-Na contains a variety of fast ionic conductors (Li_2_CO_3_ and Li_3_N), however, it lacks the support of a flexible component (organosulfide), which makes the SEI layer highly susceptible to local fracture-regeneration, accelerates the occurrence of interfacial side reactions, and leads to voltage fluctuation, the continuous depletion of active sodium, the thickening of the SEI layer and finally the severe polarization.

In contrast, LSEI-Na consists of LiF, Li_2_CO_3_, Li_3_N, Na_2_S, Na_2_SO_3_, NaF and Na_2_O components together, and its extraordinarily long cycling stability and low polarisation is the result of integration the advantages of both LiTFSI-Na and LiNO₃-Na: Firstly, Li₃N and Na₂S/Na₂SO₃ provide a fast diffusion channel of Na^+^, reducing the interfacial impedance for low polarisation (50 mV). Secondly, LSEI contains both LiF and NaF, which can improve the mechanical properties of the SEI layer. And the tough interface formed by components with high Young's modulus (NaF, LiF, and Li_2_CO_3_) and flexible organosulfide can withstand the volume expansion generated during sodium plating/stripping and resist dendrite growth. Ultimately, the synergistic effect of multiple inorganic components achieves a stable and robust SEI layer to protect the sodium metal from inhibiting side reactions during long-term cycling.


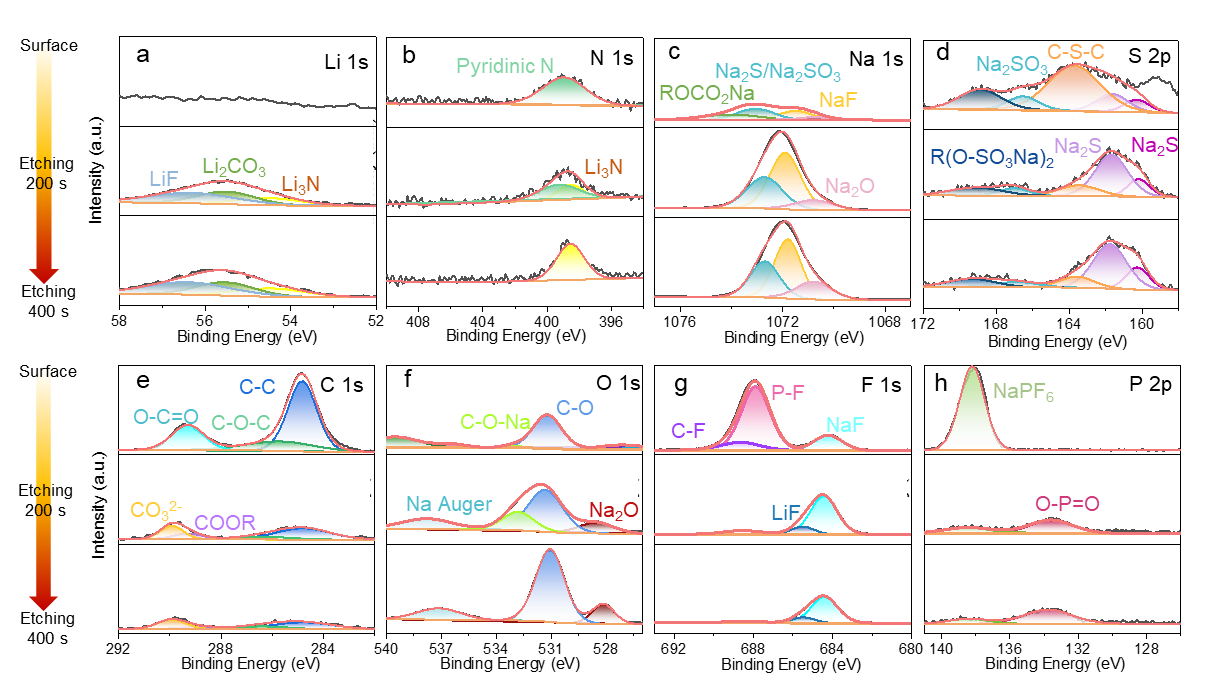


**Figure S17.** The high-resolution XPS spectra of a) Li 1s, b) N 1s, c) Na 1s, d) S 2p, e) C 1s, f) O 1s, g) F 1s and h) P 2p of LSEI-Na after 50 cycles at 10 mA cm^-2^ and 10 mAh cm^-2^ after different etching time of 0, 200, and 400 s.


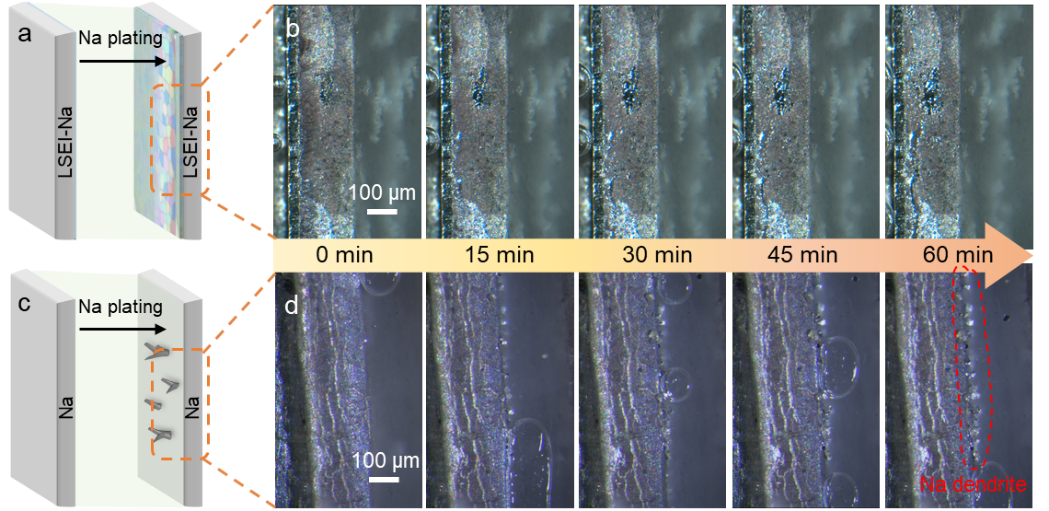


**Figure S18.** In-situ optical microscopy images of a, b) LSEI-Na and c, d) pristine Na anodes at a fixed current density of 1 mA cm^-2^.


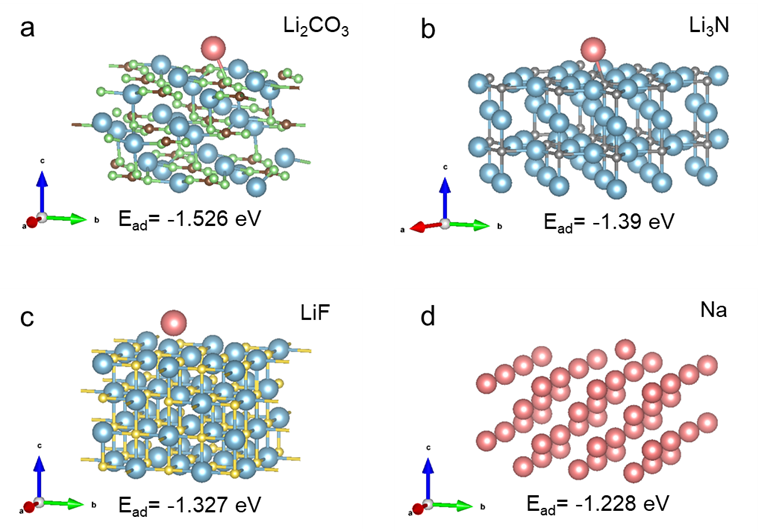


**Figure S19.** The calculated adsorption energy with Na atom for a) Li_2_CO_3_, b) Li_3_N, c) LiF and d) Na metal.


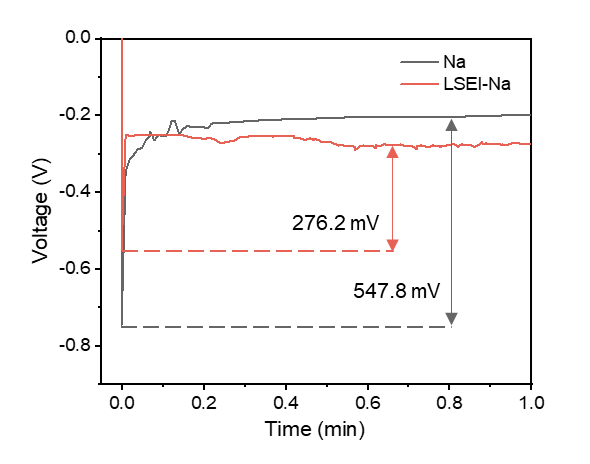


**Figure S20.** Voltage profiles of galvanostatic Na deposition on LSEI-Na and pristine Na at 30 mA cm^-2^.

**Figure S21.** In-situ electrochemical impedance spectroscopy of LSEI-Na symmetric cell at different stages under 1 mA cm^−2^ and 1 mAh cm^−2^.

**Figure S22.** The diffusion energy and corresponding diffusion paths of Na^+^ at a) Na, b) Na_2_CO_3_, c) Li_2_CO_3_, d) NaF, e) LiF, f) Na_3_N and g) Li_3_N.

**Figure S23.** The diffusion energy and corresponding diffusion paths of Na^+^ at a, b) Na_2_S and c, d) Na_2_SO_3_.


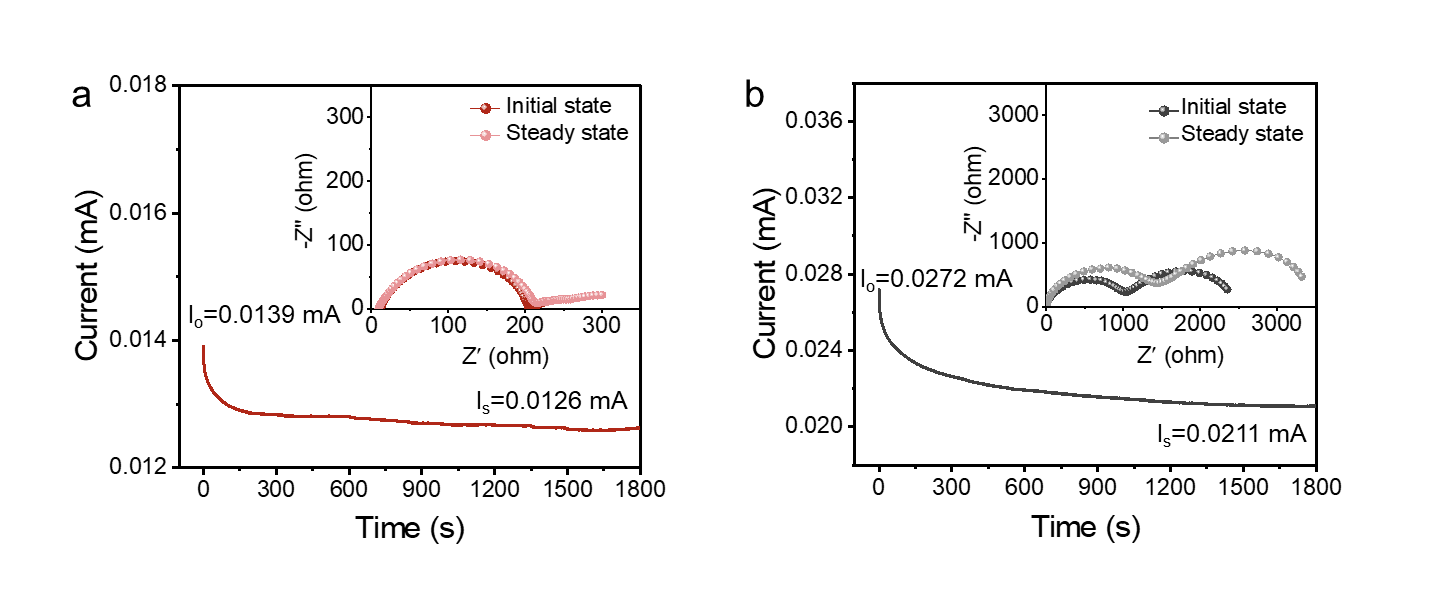


**Figure S24.** Current and impedance variations of the symmetric cells using a) LSEI-Na and b) pristine Na at room temperature with potentiostatic polarization.

**Figure S25.** The charge-discharge curves of LSEI-Na || NVP and Na || NVP full cells with different cycles at 10 C from Figure 4f.

**Figure S26.** a) The cycling performance and b) corresponding charge-discharge curves of LSEI-Na || NVP full cells at 2 C with cathode mass loadings of 8.7 mg cm^-2^.

**Figure S27.** The cycling performance of LSEI-Na || NVP full cells with cathode mass loading of 28.5 and 40.6 mg cm^-2^.


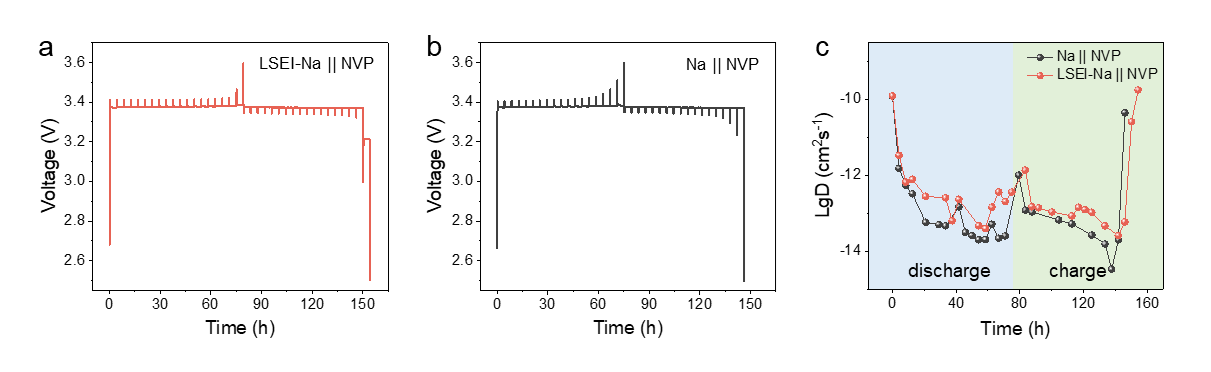


**Figure S28.** Voltage-time curves for the GITT measurement in a) LSEI-Na || NVP and b) Na || NVP full cells. c) Na^+^ diffusion coefficients during discharge/charge of LSEI-Na || NVP and Na || NVP calculated from GITT results.

**Table S1.** Comparison of the SEI layer modification strategies of previously reported works and our work.

| **Electrode** | **modification strategies** | **Ref.** |
| --- | --- | --- |
| OHTAPQ@Na | Dropwise addition method (OHTAPQ molecule was synthesized by the reported solvothermal reaction. The SEI layer was formed by dropwise addition of OHTAPQ/DEGDME solution.) | ^[5]^ |
| Na/SnSe | Rolling method (Preparation of SnSe flower-like nanocomposite by hydrothermal reaction. The heterogeneous Na/SnSe electrode was fabricated by rolling the prepared SnSe powder on the Na metal surface.) | ^[6]^ |
| NCIA | Soaking in AgClO_4_/organic solvent | ^[7]^ |
| PDBM-Na | Soaking method (The phenol-formaldehyde condensation reaction to obtain PDBM. The SEI layer was formed by immersion in PDBM/DME solution.) | ^[8]^ |
|  |  |  |
| Na@Na_2_Se/V | Rolling method (Preparation of VSe_2_ by hydrothermal reaction. The as-synthesized VSe_2_ powder was homogenously spread on the surface of fresh Na foil, followed by repeated mechanical roll-pressing.) | ^[9]^ |
|  |  |  |
| Na@g-C_3_N_4_ | Rolling transfer method (Preparation of g-C_3_N_4_, g-C_3_N_4_ slurry was then pasted onto the Cu foil surface by a facile doctor blade method and then dried, final roll transfer to sodium metal surface.) | ^[10]^ |
| IOHL-Na | Dropwise addition method (1 mmol of SnCl_2_ and 1 mmol 4-chloro-2,6-dimethylphenol were dissolved in 10 mL of THF) | ^[11]^ |
| Na_2_Te/K_2_Te | Directly painting nano-sized Te power | ^[12]^ |
| **LSEI-Na** | **Electrochemical pre-cycling method** | **This work** |

**Table S2.** Fitted *R_SEI_* and *R_ct_* values for the symmetric cells with Na, LSEI-Na and NSEI-Na at different temperatures.

| **Temperature/℃** | **Na** | | **LSEI-Na** | | **NSEI-Na** | |
| --- | --- | --- | --- | --- | --- | --- |
|  | ***R_SEI_* /Ω** | ***R_ct_* /Ω** | ***R_SEI_* /Ω** | ***R_ct_* /Ω** | ***R_SEI_* /Ω** | ***R_ct_* /Ω** |
| 30 | 989 | 1626 | 409 | 865 | 1371 | 159 |
| 35 | 793 | 1442 | 339 | 835 | 903 | 153 |
| 40 | 669 | 1338 | 280 | 767 | 682 | 145 |
| 45 | 560 | 1232 | 206 | 723 | 525 | 133 |
| 50 | 484 | 1098 | 182 | 683 | 407 | 109 |

**Table S3.** Fitted *R_SEI_* and *R_ct_* values for the LSEI-Na symmetric cells at different cycling stages.

| **State** | ***R_SEI_* (Ω)** | ***R_ct_* (Ω)** |
| --- | --- | --- |
| Discharge 20 min | 219 | 309 |
| Discharge 40 min | 210 | 365 |
| Discharge 60 min | 210 | 374 |
| Charge 20 min | 218 | 236 |
| Charge 40 min | 190 | 218 |
| Charge 60 min | 175 | 180 |

**Table S4.** The fitted R_0_ and R_S_ values for the symmetrical cells with the different anodes.

| **Anode** | ***R_0_* (Ω)** | ***R_S_* (Ω)** |
| --- | --- | --- |
| LSEI-Na | 193 | 199 |
| pristine Na | 2563 | 3630 |

**Table S5.** Comparison of the cycling performances of symmetrical cells of previously reported works and our work.

| **Electrode** | **Current density (mA cm^-2^)** | | **Areal capacity (mAh cm^-2^)** | | **cycling performances** | **Electrolyte** | **Ref.** |
| --- | --- | --- | --- | --- | --- | --- | --- |
| w/NaBr | 1 | 0.5 | | 200 mV, 250 h | | 1M NaPF_6_ EC/ PC | ^[13]^ |
| Na-3DHS | 1 | 1 | | ~90 mV, 350 h | | 1 M NaClO_4_ in EC/DEC with 5% FEC | ^[14]^ |
| Polished Na | 3 | 1 | | ~25 mV, 800 h | | 1M NaOTf in diglyme | ^[15]^ |
|  | 5 | 1 | | ~50 mV, 400 h | |  |  |
| Cu_3_P@Cu | 1 | 1 | | ~3 mV, 2400 h | | 1M NaPF_6_ in diglyme | ^[16]^ |
|  | 2 | 2 | | ~9 mV, 2000 h | |  |  |
| FCTF/Na | 2 | 1 | | ~25 mV, 2500 h | | 1M NaPF_6_ in diglyme | ^[17]^ |
|  | 4 | 9.5 | | ~30 mV, 1300 h | |  |  |
| Na@25Al_2_O_3_ | 3 | 1 | | 20 mV, 500 h | | 1 M NaCF_3_SO_3_ dissolved in DEGDME | ^[18]^ |
| Na/NSCNT | 1 | 1 | | ~50 mV, 500 h | | 1 M sodium triflate in diglyme | ^[19]^ |
| mPG-12@PP | 1 | 1 | | ~50 mV, 2000 h | | 1M NaPF_6_ in diglyme | ^[12]^ |
|  | 5 | 5 | | ~20 mV, 1700 h | |  |  |
|  | 10 | 10 | | ~20 mV, 1400 h | |  |  |
| HCOONa-Na | 2 | 1 | | ~45 mV, 2250 h | | 1M NaPF_6_ in diglyme | ^[20]^ |
| **LSEI-Na** | **3** | **1** | | **~50 mV, 5900 h** | | **1M NaPF_6_ in diglyme** | **This work** |
|  | **3** | **3** | | **~40 mV, 2400 h** | |  |  |
|  | **10** | **10** | | **~125 mV, 1800 h** | |  |  |
|  | **30** | **30** | | **~160 mV, 500 h** | |  |  |

**Table S6.** The comparison of the electrochemical performance of NVP-based full cells between reported works and this work.

| **Anode** | **Current density** | **Discharge specific capacity (mAh g^-1^)** | **Capacity retention/Cycle number** | **Active mass loading (mg cm^-2^)** | **Ref.** |
| --- | --- | --- | --- | --- | --- |
| mPG-12@PP | 2 C | - | 90% / 500 cycles | 1.0 | ^[12]^ |
| SnCl_4_@Na-rGO | 1 C | 106 | 92.6% / 600 cycles | 1.2-1.5 | ^[21]^ |
| Na_2_S/V/Na | 10 C | 90 | 88.2% / 1400 cycles | 6.0 | ^[22]^ |
| At-Sn@HCN/Na | 2 C | ~82 | 800 cycles | 3.0 | ^[23]^ |
|  | - | 81.4 | 93% / 100 cycles | 7.0 (N/P=1.2) |  |
| A-SnS-G@Na | 0.4 C | 73.8 | 400 cycles | 6.0 | ^[24]^ |
| Na-3DHS | 10 C | ~60 | 900 cycles | 1.61 | ^[14]^ |
| MgF_2_@RGO | 1 C | 79.88 | 91.3% / 200 cycles | 2.0 | ^[25]^ |
|  | 0.5C | ~110 | 30 cycles | 15.0 (N/P=2.5) |  |
| HCOONa-Na | 2 C | 101.4 | 96.7% / 800 cycles | 10.0 | ^[20]^ |
| NCIA | 0.3 C | 112 | 97.5% / 53 cycles | 20.0 | ^[7]^ |
| MgF_2_@RGO | 0.5 C | 108 | 30 cycles | 15.0 (N/P=2.5) | ^[25]^ |
| **LSEI-Na** | **2 C** | **95.0** | **91.3% / 1300 cycles** | **3.8** | **This work** |
|  | **10 C** | **94.7** | **92.9% / 1600 cycles** | **3.5** |  |
|  | **2 C** | **88.9** | **91.9% / 800 cycles** | **8.7** |  |
|  | **2 C** | **90.4** | **101% / 280 cycles** | **12.8** |  |
|  | **1 C** | **102.1** | **98.7% / 90 cycles** | **28.5** |  |
|  | **0.5 C** | **101.2** | **97.9% / 120 cycles** | **39.3** |  |
|  | **1 C** | **99.6** | **96.9% / 240 cycles** | **8.1 (N/P=1.21)** |  |

**References**

[1] aY. Li, F. Wu, Y. Li, M. Liu, X. Feng, Y. Bai, C. Wu, *Chem. Soc. Rev.* **2022**, *51*, 4484-4536; bZ. Lin, Q. Xia, W. Wang, W. Li, S. Chou, *InfoMat* **2019**, *1*, 376-389; cJ. Zhang, D. W. Wang, W. Lv, L. Qin, S. Niu, S. Zhang, T. Cao, F. Kang, Q. H. Yang, *Adv. Energy Mater.* **2018**, *8*, 1801361; dJ. Zhang, D.-W. Wang, W. Lv, S. Zhang, Q. Liang, D. Zheng, F. Kang, Q.-H. Yang, *Energy Environ. Sci.* **2017**, *10*, 370-376.

[2] aG. Kresse, J. Furthmüller, *Phys. Rev. B* **1996**, *54*, 11169; bG. Kresse, D. Joubert, *Phys. Rev. B* **1999**, *59*, 1758.

[3] G. Henkelman, B. P. Uberuaga, H. Jónsson, *J. Chem. Phys.* **2000**, *113*, 9901-9904.

[4] G. Wang, L. Peng, K. Li, L. Zhu, J. Zhou, N. Miao, Z. Sun, *Comput. Mater. Sci.* **2021**, *186*, 110064.

[5] Z. Shen, Z. Bo, R. Shi, L. Liu, H. Li, X. Zhou, J. Wang, Y. Zhao, L. Li, *Adv. Funct. Mater.* **2025**, 202420573.

[6] L. Cao, J. Guo, Y. Feng, Y. Li, Y. Qiu, W. Zhu, Y. Tan, C. Sun, X. Rui, H. Geng, *Adv. Funct. Mater.* **2024**, 202313962.

[7] J. Xie, Z. Li, X. Zheng, F. Tian, D. Lei, C. Wang, *Adv. Funct. Mater.* **2024**, *34*, 2315309.

[8] Q. Yue, Z. Shen, R. Shi, Q. Zhang, L. Liu, D. Shi, S. Jiao, Y. Zhao, *ACS Energy Letters* **2024**, *9*, 2265-2275.

[9] X. Xia, S. Xu, F. Tang, Y. Yao, L. Wang, L. Liu, S. He, Y. Yang, W. Sun, C. Xu, Y. Feng, H. Pan, X. Rui, Y. Yu, *Adv. Mater.* **2023**, *35*, e2209511.

[10] J. Wang, Z. Liu, B. Qu, Z. Li, Y. Zhang, C. Xu, Z. Chen, Q. Xie, M.-S. Wang, M. Lu, J. Zhao, J. Wang, D.-L. Peng, F. Pan, Y.-S. Hu, *Energy Storage Mater.* **2023**, *59*, 102793.

[11] P. Liu, L. Miao, Z. Sun, X. Chen, Y. Si, Q. Wang, L. Jiao, *Angew. Chem., Int. Ed.* **2023**, *62*, e202312413

[12] J. Qin, H. Shi, K. Huang, P. Lu, P. Wen, F. Xing, B. Yang, M. Ye, Y. Yu, Z.-S. Wu, *Nat. Commun.* **2021**, *12*, 5786.

[13] S. Choudhury, S. Wei, Y. Ozhabes, D. Gunceler, M. J. Zachman, Z. Tu, J. H. Shin, P. Nath, A. Agrawal, L. F. Kourkoutis, T. A. Arias, L. A. Archer, *Nat. Commun.* **2017**, *8*, 898.

[14] M. Zhu, S. Li, B. Li, Y. Gong, Z. Du, S. Yang, *Sci. Adv.* **2019**, *5*, eaau6264.

[15] Y. Gu, W.-W. Wang, Y.-J. Li, Q.-H. Wu, S. Tang, J.-W. Yan, M.-S. Zheng, D.-Y. Wu, C.-H. Fan, W.-Q. Hu, Z.-B. Chen, Y. Fang, Q.-H. Zhang, Q.-F. Dong, B.-W. Mao, *Nat. Commun.* **2018**, *9*, 1339.

[16] W. Zhang, J. Zheng, Z. Ren, J. Wang, J. Luo, Y. Wang, X. Tao, T. Liu, *Adv. Mater.* **2024**, *36*, 2310347.

[17] R. Zhuang, X. Zhang, C. Qu, X. Xu, J. Yang, Q. Ye, Z. Liu, S. Kaskel, F. Xu, H. Wang, *Sci. Adv.* **2023**, *9*, eadh8060.

[18] Y. Zhao, L. V. Goncharova, A. Lushington, Q. Sun, H. Yadegari, B. Wang, W. Xiao, R. Li, X. Sun, *Adv. Mater.* **2017**, *29*, 1606663.

[19] B. Sun, P. Li, J. Zhang, D. Wang, P. Munroe, C. Wang, P. H. L. Notten, G. Wang, *Adv. Mater.* **2018**, *30*, 1801334.

[20] C. Wang, Y. Zheng, Z. N. Chen, R. Zhang, W. He, K. Li, S. Yan, J. Cui, X. Fang, J. Yan, G. Xu, D. Peng, B. Ren, N. Zheng, *Adv. Energy Mater.* **2023**, *13*, 2204125.

[21] X. Lai, Z. Xu, X. Yang, Q. Ke, Q. Xu, Z. Wang, Y. Lu, Y. Qiu, *Adv. Energy Mater.* **2022**, *12*, 2103540.

[22] Y. Jiang, Y. Yang, F. Ling, G. Lu, F. Huang, X. Tao, S. Wu, X. Cheng, F. Liu, D. Li, H. Yang, Y. Yao, P. Shi, Q. Chen, X. Rui, Y. Yu, *Adv. Mater.* **2022**, *34*, e2109439.

[23] F. Xu, C. Qu, Q. Lu, J. Meng, X. Zhang, X. Xu, Y. Qiu, B. Ding, J. Yang, F. Cao, P. Yang, G. Jiang, S. Kaskel, J. Ma, L. Li, X. Zhang, H. Wang, *Sci. Adv.* **2022**, *8*, eabm7489.

[24] W. Liu, Z. Chen, Z. Zhang, P. Jiang, Y. Chen, E. Paek, Y. Wang, D. Mitlin, *Energy Environ. Sci.* **2021**, *14*, 382-395.

[25] L. Zhao, Z. Hu, Z. Huang, Y. Tao, W. H. Lai, A. Zhao, Q. Liu, J. Peng, Y. Lei, Y. X. Wang, Y. Cao, C. Wu, S. L. Chou, H. K. Liu, S. X. Dou, *Adv. Energy Mater.* **2022**, *12*, 2200990.
